# Characterization of New Gambierones Produced by *Gambierdiscus balechii* 1123M1M10

**DOI:** 10.3390/md21010003

**Published:** 2022-12-21

**Authors:** Xiaowan Liu, Yihan Ma, Jiajun Wu, Qizhao Yin, Pengbin Wang, Jingyi Zhu, Leo Lai Chan, Bin Wu

**Affiliations:** 1 The State Key Laboratory of Marine Pollution, Department of Biomedical Sciences, City University of Hong Kong, Hong Kong SAR 999077, China; 2 Ocean College, Zhejiang University, Zhoushan 321000, China; 3 Shenzhen Key Laboratory for the Sustainable Use of Marine Biodiversity, Research Centre for the Oceans and Human Health, City University of Hong Kong Shenzhen Research Institute, Shenzhen 518057, China; 4 Key Laboratory of Marine Ecosystem Dynamics, Second Institute of Oceanography, Ministry of Natural Resources, Hangzhou 310012, China; 5 The Fourth Institute of Oceanography, Ministry of Natural Resources, Beihai 536000, China

**Keywords:** *Gambierdiscus balechii*, gambierone, dihydro-44-methylgambierone, dehydroxy-44-methylgambierone, desulfo-hydroxyl gambierone

## Abstract

The benthic dinoflagellate genus *Gambierdiscus* is the primary producer of toxins responsible for ciguatera poisoning (CP), a food intoxication endemic in tropical and subtropical areas of the world. We used high-performance liquid chromatography tandem high-resolution mass spectrometry (HPLC-HRMS) to investigate the toxin profile of *Gambierdiscus balechii* 1123M1M10, which was obtained from Marakei Island (2°01′N, 173°15′E), Republic of Kiribati, located in the central Pacific Ocean. Four new gambierone analogues including 12,13-dihydro-44-methylgambierone, 38-dehydroxy-12,13-dihydro-44-methylgambierone, 38-dehydroxy-44-methylgambierone, and desulfo-hydroxyl gambierone, and two known compounds, gambierone and 44-methylgambierone, were proposed by analyzing their fragmentation behaviors and pathways. Our findings provide new insights into the toxin profile of *Gambierdiscus balechii* 1123M1M10, which can be used as a biomarker for species identification, and lay the foundation for further toxin isolation and bioactivity studies of gambierones.

## 1. Introduction

*Gambierdiscus* is a genus of marine autotrophic epi-benthic dinoflagellate that grows on the surface of macroalgae, corals, and sand grains [1,2,3]. This dinoflagellate has gained scientists’ attention due to its production of ladder-shaped polyether toxins, including ciguatoxins (CTXs) [4,5], maitotoxins (MTXs) [6,7,8], gambierones [9,10,11], gambierol [9], gambieric acids [10], and gambieroxide [11]. Most of these toxins can bioaccumulate in the food chain, and the consumption of fish or shellfish contaminated with these toxins can cause ciguatera food poisoning (CFP), which is the most common non-microbial foodborne illness occurring in the tropical and subtropical regions of the world [12]. Patients with CFP may suffer from gastrointestinal, cardiological, and long-lasting neurological symptoms [13]. It is estimated that nearly 50,000 people are affected by CFP annually [14]. However, no effective CFP prevention and treatment strategy has been presented thus far, which is attributable to a lack of pure toxins and a poor understanding of their toxicity. Although these toxins are notoriously toxic, they have potential medicinal value because of their desirable biological activities. For instance, gambieric acids show significant antifungal properties [10], while gambierol inhibits voltage-gated potassium channels [15,16]. Maitotoxins (MTXs) enhance calcium ion influx across cell membranes [17], and CTXs affect various sodium-dependent mechanisms [18]. Thus, the identification of novel toxins from *Gambierdiscus* spp. could promote the development of toxin detection methods and pharmacological applications.

The toxicity levels of *Gambierdiscus* show marked variation among species, and even within species [19]. Although the underlying biosynthesis of toxins remains unclear, several studies have illustrated that environmental factors including temperature, light, salinity, pH, and bacterial communities, can affect toxin production in *Gambierdiscus* [20,21,22]. Moreover, studies of polyether chemical structures suggest that the synthesis of polyketides mediated by polyketide synthase is likely involved in the biosynthetic process of these toxins [23]. To date, there are 18 *Gambierdiscus* species that have been identified by morphology and molecular methods [24]. Among them, the species *G. balechii* is less studied, and its toxin profile has not been adequately investigated. Only a few studies have reported the toxicology and toxins of this species. For instance, the strains originating from the Kiribati Islands and Malaysia have shown CTX-like toxicity, and the strain from Indonesia showed CTX-like and MTX-like toxicities in the neuroblastoma N2A cytotoxicity assay [19,25]. The strain from the Philippines was confirmed to produce 44-methylgambierone by LC-MS/MS analysis [26]. Since this species is a causative organism of CFP, and the degree to which individual strains vary in toxicity and toxin profile, further investigations are needed to analyze its toxin components. 

It is reported that CTXs and MTXs are major toxins involved in CFP [27,28] and are widely noticed and studied. However, nuclear magnetic resonance (NMR) and high-performance liquid chromatography tandem mass spectrometry (HPLC-MS) analyses have shown that only two species (*G. toxicus* and *G. polynesiensis*) are the producers of CTXs [4,29,30,31,32,33], while five species (*G. toxicus*, *G. australes*, *G. cheloniae*, *G. honu*, and *G. excentricus*) can produce MTXs [7,34,35,36]. In contrast to CTXs and MTXs, gambierone and its analogues are frequently found in *Gambierdiscus* species. For instance, except for three species (*G. scabrosus*, *G. carolinianus*, and *G. jejuensis*) with no information on NMR or HPLC-MS analyses of toxins, all reported *Gambierdiscus* species have been confirmed to produce gambierones [6,24,26,33,37,38,39,40,41,42,43,44]. Although gambierone and 44-methylgambierone are less toxic than CTXs, their extensive distribution proves their vital role [24,45]. Moreover, there are only four gambireones reported hitherto, including gambierone, 44-methylgambierone, and two partially identified sulfo-gambierones [8,39,40]. A lack of structural information and insufficient samples have limited the comprehensive evaluation of these toxins. Thus, there is an urgent need to decipher novel gambierones for a further understanding of their roles in CFP and potential applications in pharmacology.

In this study, we applied high-performance liquid high-resolution mass spectrometry (HPLC-HRMS) analyses to investigate the toxin profile of *G. balechii* 1123M1M10, which was obtained from Marakei Island, Republic of Kiribati, located in the central Pacific Ocean [19]. We identified six toxins, including four new toxins named 12,13-dihydro-44-methylgambierone, 38-dehydroxy-12,13-dihydro-44-methylgambierone, 38-dehydroxy-44-methylgambierone, and desulfo-hydroxyl gambierone, and two known compounds, gambierone and 44-methylgambierone from the *G. balechii* 1123M1M10. 

## 2. Results and Discussion

### 2.1. Investigation of Toxin Profile in Gambierdiscus balechii 1123M1M10

**Figure 1:**
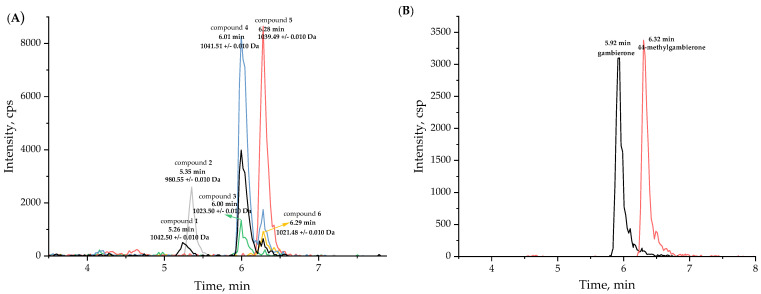
(**A**) Extraction ion chromatograms (XICs) of putative gambierone analogues in fraction 3, and (**B**) XICs of standards of gambierone (5.92 min) at 345 ng/mL, and 44-methylgambierone (6.32 min) at 315 ng/mL, using IDA method in positive ESI mode.

Some studies and our previous report have demonstrated that *G. balechii* 1123M1M10 has CTX-like toxicity, suggesting that it may be the producer of CTXs or gambierones [8,41,46]. To characterize the toxin profile of *G. balechii* 1123M1M10, an untargeted metabolomics study was conducted using electrospray ionization time-of-flight mass spectrometry (ESI-TOF-MS) operated with an information-dependent acquisition (IDA) method. The analysis performed in positive mode yielded more chemical information than in the negative mode, and was therefore used for further investigation. It was reported that the general fragmentation pathways of ladder polyether compounds were the dissociation of one covalent C-C bond, along with one or two covalent C-O bonds, and the typical feature of their fragmentations was the consecutive loss of H_2_O (18 Da), since the dissociation of polarized C-O bonds occurs more easily than C-C bonds [47]. According to the analyses of MS^2^ fragments—including the fragment of the side chain in the ring I *m/z* 109, specific fragments *m/z* 233 or *m/z* 219, sulfate loss (*m/z* 959 or 945), and successive water losses—six putative gambierone analogues were found from fractions 3 to 8 from a total of 18 fractions obtained from the Sephadex LH-20 chromatography performed in this study. Four of them were tentatively proposed as novel compounds (desulfo-hydroxyl gambierone (**2**), 38-dehydroxy-12,13-dihydro-44-methylgambierone (**3**), 12,13-dihydro-44-methylgambierone (**4**) and 38-dehydroxy-44-methylgambierone (**6**)) and were eluted at 5.35 min, 6.00 min, 6.01 min, and 6.29 min, respectively (Figure 1A). The other two compounds were proposed as gambierone (**1**) and 44-methylgambierone (**5**), based on the comparison of the fragmentation behaviors and retention times with the published data and standards [40,48]. The retention time of putative 44-methylgambierone was 6.28 min, consistent with the standard, while the putative gambierone (retention time: 5.26 min) was eluted earlier than the standard (retention time: 5.92 min) (Figure 1). Under the same analysis conditions, the putative gambierone and gambierone standard had similar mass spectra and the same specific product ions, indicating that they may have the same structures with different configurations. Of the six gambierone analogues, four of them were not detected by the IDA method in the negative ESI mode due to their low concentration in the sample. In the ESI (−)- MS^2^ spectra, the deprotonated ions [M − H]^−^ and hydrogen-sulfate fragments [HOSO_3_]^−^ at *m/z* 1039.4908 and 96.9598 in compound **4**, and 1037.4748 and 96.9599 in compound **5,** were observed, showing the same pattern as the standards (Figure S2).

**Figure 2:**
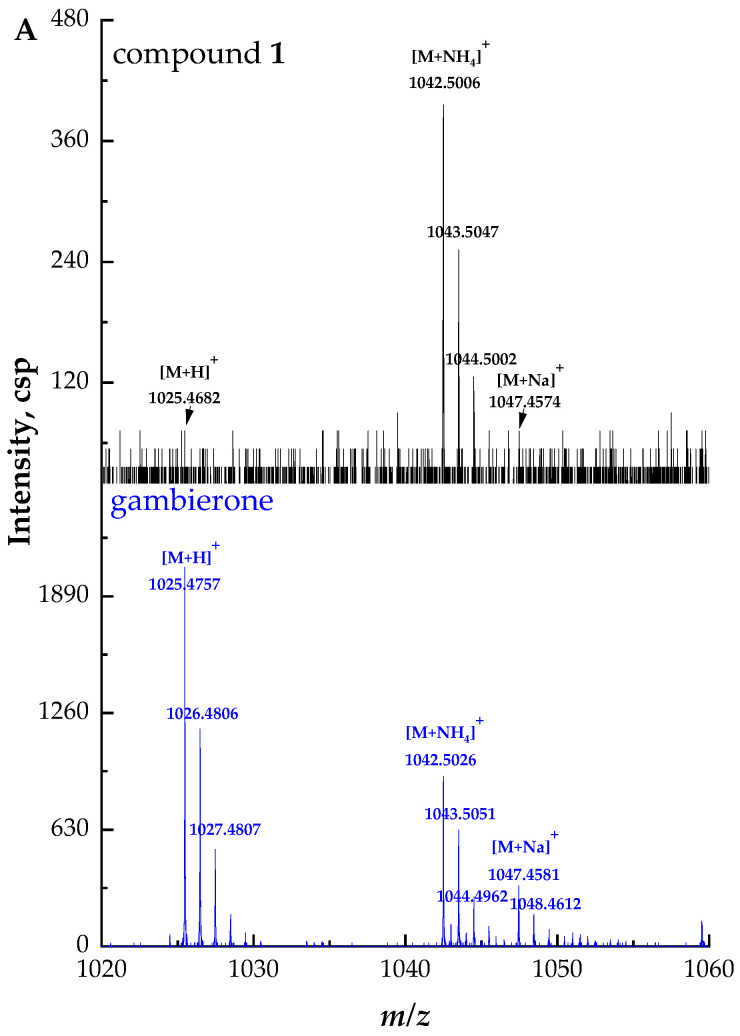

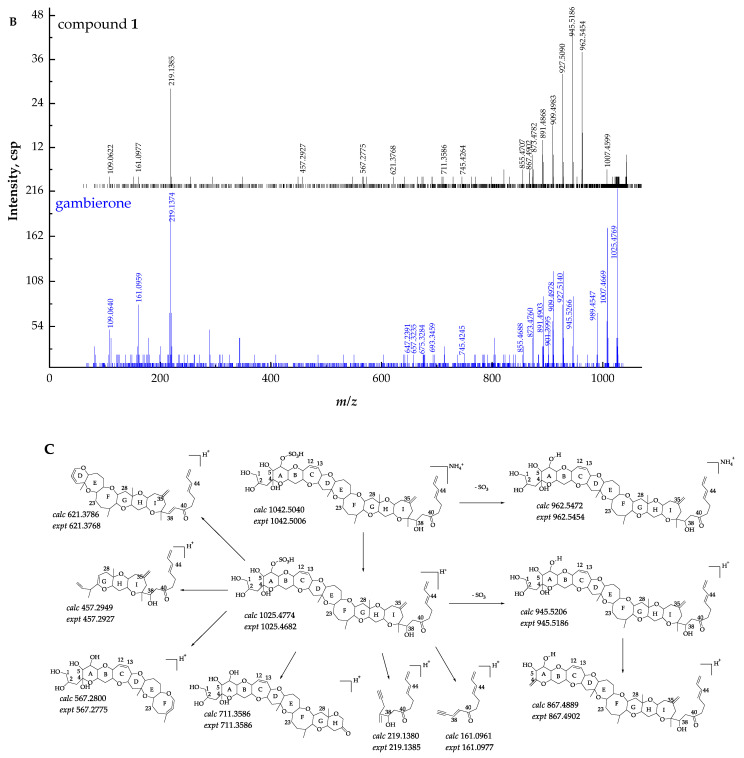
(**A**) Full-scan mass spectra (MS^1^) of compound **1** and gambierone standard, (**B**) fragment ion spectra (MS^2^, MS/MS) of [M + NH_4_]^+^ ion of compound **1** and [M + H]^+^ ion of gambierone standard, (**C**) fragmentation pathways proposed for compound **1**.

Compound **1** ([M + NH_4_]^+^ *m/z* 1042.5006, C_51_H_80_NO_19_S^+^, Δ 3.3 ppm) was considered to have the same structure with different configurations as gambierone according to the comparison of their retention times and fragment ions. The full-scan mass spectrum of compound **1** showed proton, ammonium, and sodium adduct precursor ions at *m/z* 1025, 1042, and 1047, respectively, which corresponded with the standard but with different ion ratios (Figure 2A). However, compound **1** showed similar product ions with gambierone in the MS^2^ spectra (Figure 2B). The proposed fragmentation pathways of this compound are discussed below (Figure 2C). The fragmentation of the ammonium loss plus the water loss produced the proton adduct ion at *m/z* 1025 and the pseudo-molecular ion [M + H − H_2_O]^+^ at *m/z* 1007, which is consistent with that of the standard under the same condition. The product ion at *m/z* 962, which was not present in the MS^2^ spectrum of the standard, was proposed as the pseudo-molecular ion [M + NH_4_ − SO_3_]^+^ with a mass difference (∆ppm) of 1.9 ppm. The precursor ion at *m/z* 1025 yielded the product ion at *m/z* 945, which was attributed to the elimination of the neutral loss of SO_3_ (80 Da). Five product ions at *m/z* 927, 909, 891, 873, and 855 were generated by the loss of H_2_O (18 Da), 2 H_2_O (36 Da), 3 H_2_O (54 Da), 4 H_2_O (72 Da), and 5 H_2_O (90 Da), respectively, from the ion at *m/z* 945, consistent with the gambierone standard and reported MS data [48]. The product ion at *m/z* 867 was generated by the cleavage of the right-side chain and the broken C-C bond was between C-2 and C-3. In addition to the cleavage of the side chain, the common cleavage of ring I was also observed in compound **1**. The product ion at *m/z* 711 was formed by the cleavage of one covalent C-C bond and one C-O bond in ring I (C_33_-C_34_ and C_37_-O), plus the further loss of SO_3_ (80 Da). The fragment ion at *m/z* 219 was produced by the cleavage of the C-O bond (C-37) along with the C-C bond between C-34 and C-35. The product ion at *m/z* 161 was formed by the cleavage of the C-O bond located at the C-37 and C-C bond (C-36 and C-37), plus the further loss of H_2_O (18 Da). Furthermore, the cleavage of the ring G (C_27_-C_28_ and C_26_-O) plus the loss of SO_3_ (80 Da) generated the fragment ion at *m/z* 567. The product ion at *m/z* 457 was formed by the dissociation of one covalent C-C bond (C-22 and C-23) and one C-O bond (C-27) in the ring F, and the elimination of SO_3_ (80 Da). The product ion at *m/z* 621 was formed by the dissociation of one covalent C-C bond between C-13 and C-14, and one C-O bond located at C-15 in the ring C. The fragmentation pathways were proposed based on MS^2^ data, and their mass differences (∆ppm) were less than 10 ppm (Table S1).

**Figure 3:**
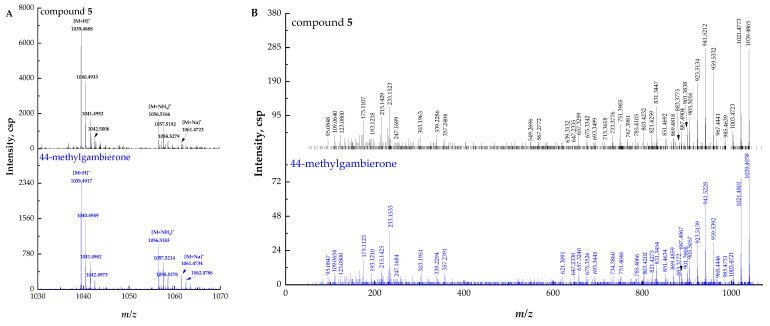

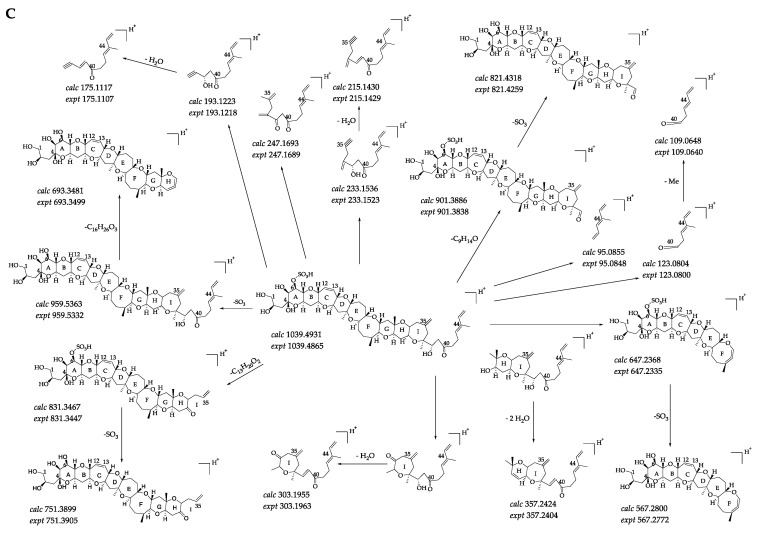
(**A**) Full-scan mass spectra (MS^1^) of compound **5** and 44-methylgambierone standard, (**B**) fragment ion spectra (MS^2^, MS/MS) of [M + H]^+^ ions of compound **5** and 44-methylgambierone standard, (**C**) fragmentation pathways proposed for [M + H]^+^ ion of compound **5**.

Compound **5** ([M + H]^+^ *m/z* 1039.4865, C_52_H_79_O_19_S^+^, Δ 6.3 ppm) could unambiguously be identified as 44-methylgambierone by comparing the retention times and mass spectra with the standard and analyzing the proposed fragmentation pathways. The full-scan mass spectrum of compound **5** showed proton, ammonium, and sodium adduct precursor ions at *m/z* 1039, 1056, and 1061, respectively, which were consistent with the reference standard (Figure 3A). Under the same MS^2^ condition, compound **5** showed almost the same MS^2^ ions as 44-methylgambierone (Figure 3B). The proposed fragmentation pathways of the putative 44-methylgambierone were discussed below (Figure 3C). The precursor ion yielded product ions at *m/z* 1021, 1003, 985, and 967, which were attributed to the elimination of the sequential neutral loss of H_2_O (18 Da) from the precursor ion at *m/z* 1039. The product ion at *m/z* 959 was formed by the neutral loss of SO_3_ (80 Da) from the [M + H]^+^ precursor ion; then, the elimination of H_2_O (18 Da), 2 H_2_O (36 Da), 3 H_2_O (54 Da), 4 H_2_O (72 Da), 5 H_2_O (90 Da), and 6 H_2_O (108 Da) yielded product ions at *m*/*z* 941, 923, 905, 887, 869, and 851, respectively. Except for the neutral losses, there were many fragments obtained by the cleavage of the right-side chain. For instance, the fragment ion at *m/z* 901 was achieved with the cleavage of the C-C bond between C-38 and C-39, and then the elimination of H_2_O (18 Da) produced the product ion at *m/z* 883, while the elimination of SO_3_ (80 Da) plus the further sequential loss of H_2_O (18 Da) produced fragment ions at *m/z* 821, 803, 785, and 767. The small fragment ion at *m/z* 123 was formed by the cleavage of the C-C bond between C-39 and C-40, and then the elimination of the methyl group produced the fragment ion at *m/z* 109. The product ion at *m/z* 95 was generated by the breaking of the C-C bond located at C-40 and C-41. In addition, the cleavage of the ring I was also observed in this study. The product ion at *m/z* 693 was formed from the fragment ion at *m/z* 959 by the elimination of C_16_H_26_O_3_ (266 Da), which corresponded to the dissociation of one covalent C-C bond between C-33 and C-34, and one C-O bond located at C-32 in the ring I. The sequential loss of H_2_O (18 Da) from the product ion at *m/z* 693 produced fragment ions at *m/z* 675, 657, and 639. Fragment ion at *m/z* 831 was also obtained by the cleavage of the ring I but at different sites (C_35_-C_36_ and C_37_-O), and then the elimination of SO_3_ (80 Da) plus the further sequential loss of H_2_O (18 Da) produced fragment ions at *m/z* 751, 733, and 715, respectively. Furthermore, the cleavage of ring I generated five small fragments at *m/z* 247, 233, 215, 193, and 175, which have been commonly observed in previous studies [40,48]. The fragment ion at *m/z* 303 was obtained by the cleavage of one covalent C-C bond (C-30 and C-31) and one C-O bond (C-29) in the ring H plus the neutral loss of H_2_O (18 Da). Furthermore, the cleavage of the ring G generated two moieties. Then, the loss of SO_3_ (80 Da) and sequential losses of H_2_O (18 Da) from the left moiety at *m/z* 647 generated two fragment ions at *m/z* 567 and 549, while the elimination of two and three H_2_O (18 Da) from the right moiety produced the fragment ions at *m/z* 357 and 339. The fragmentation pathways were proposed based on MS^2^ data, and their mass differences (∆ppm) were less than 10 ppm (Table S2).

### 2.2. Identification and Characterization of New Gambierone Analogues

#### 2.2.1. Fragmentation Pathways of 12,13-Dihydro-44-Methylgambierone

**Figure 4:**
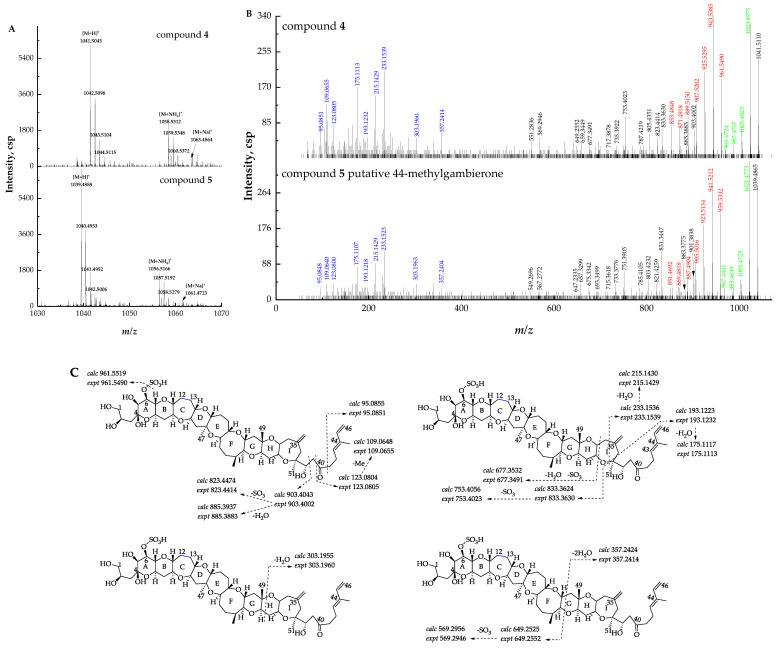
(**A**) Full-scan mass spectra (MS^1^) of compounds **4** and **5**, (**B**) fragment ion spectra (MS^2^, MS/MS) of [M + H]^+^ ions of compounds **4** and **5**. The fragment ions that were identical to putative 44-methylgambierone are shown in blue, and fragment ions with 2.0 Da mass differences in both compounds are shown in black, red, and green. (**C**) Fragmentation proposed for compound **4**.

Compound **4** ([M + H]^+^ *m/z* 1041.5110, C_52_H_81_O_19_S^+^, Δ −2.2 ppm) was proposed to be 12,13-dihydro-44-methylgambierone based on the comparison of the retention times, precursor ions, fragment ions, and ion ratios with those of compound **5**. The mass differences of specific ions of both compounds were 2.0 Da, including three types of precursor ions (Figure 4A), fragment ions yielded by sequential H_2_O (18 Da) losses from the precursor ions, and product ions yielded by the loss of SO_3_ (80 Da) plus sequential losses of H_2_O (18 Da) from the precursor ions (Figure 4B), suggesting the existence of two additional hydrogens in the structure of compound **4**. The proposed fragmentation pathways of the above-mentioned compound **5** showed that the cleavage of the right-side chain, I, H, and G rings yielded product ions at *m/z* 95, 123, 109, 233, 215, 193, 175, 303, and 357. These fragment ions, which were diagnostically related to the characterization of right-side moieties, were also observed in compound **4** (Figure 4C), suggesting that the two compounds shared the same structure at the right side of ring G and the additional hydrogens were likely located at the left side of ring G. Moreover, the fragmentation of putative 44-methylgambierone showed that the cleavage of the C-C bond (C-38 and C-39) at the right-side chain plus the further elimination of H_2_O (18 Da) and SO_3_ (80 Da) produced fragment ions at *m/z* 901, 883, 821, 803, and 785. The same fragment patterns were observed in compound **4,** yielding product ions at *m/z* 903, 885, 823, 805, and 787, which were 2.0 Da higher than those of the fragment ions of putative 44-methylgambierone. The same phenomenon was observed in other left-side moieties produced by the cleavage of rings I, H, and G (Figure 4C), which supported our hypothesis that two additional hydrogens were located at the left side of ring G. Since both compounds were very similar in ion ratios and in-source fragmentation, two additional hydrogens were proposed to be located at C-12 and C-13 which ensured the intactness of the whole ladder-shaped backbone. Compound **4** was thus proposed to be a new gambierone analogue named 12,13-dihydro-44-methylgambierone. The proposed attributions of the ion formulas, along with mass differences, are presented in Table S3. 

#### 2.2.2. Fragmentation Pathways of 38-Dehydroxy-12,13-Dihydro-44-Methylgambierone

**Figure 5:**
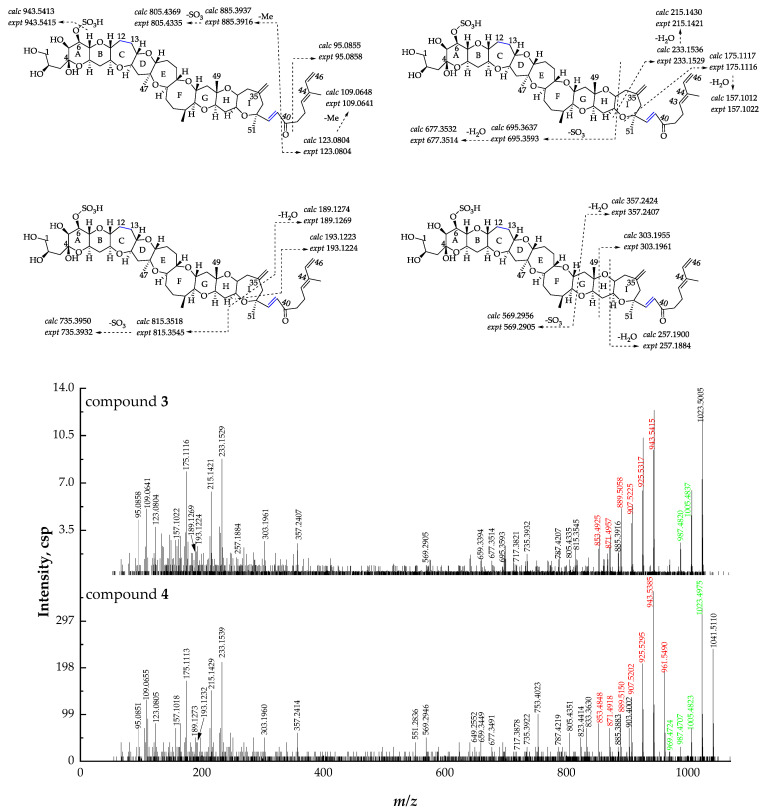
Fragment ion spectra (MS^2^, MS/MS) of [M + H]^+^ ions of compounds **3** and **4**, and fragmentation proposed for compound **3**. The product ions formed by H_2_O losses from precursor ions are shown in green, and the ions formed by the SO_3_ loss plus H_2_O losses from precursor ions are shown in red.

By comparing the retention time and MS data with compound **4,** compound **3** ([M + H]^+^ *m/z* 1023.5005, C_52_H_79_O_18_S^+^, Δ -2.2 ppm) was proposed to be 38-dehydroxy-12,13-dihydro-44-methylgambierone (Figure 5). The precursor ion [M + H]^+^ at *m/z* 1023, the neutral loss of SO_3_, and sequential losses of H_2_O were observed using the IDA mode, indicating that compound **3** was a gambierone analogue. Because of the low concentration of this compound, the MS^2^ spectrum obtained via the IDA mode was not suitable for further fragmentation analysis. To solve this problem, the high-resolution multiple reaction monitoring (MRM^HR^) acquisition method was applied to analyze compound **3**. The MRM^HR^ workflow is similar to the MRM of the QTRAP system, which uses quadrupole filters to reduce noise and increase selectivity, but the third quadrupole is replaced by TOF, and all fragments generated by a selected precursor can be analyzed in this system. The mass differences of precursor ions and product ions produced by the first SO_3_ loss from the precursor ions between compounds **3** and **4** were 18 Da, indicating the presence of dehydroxylation in compound **3**. The observation of fragment ions at *m/z* 123, 109, and 95 in the MS^2^ spectra of compounds **3** and **4** indicated that both compounds had the same structure from C-40 to C-46. The product ions at *m/z* 815, 735, 695, 677, and 569 were formed by the cleavage of rings I and G, suggesting the same structure as compound **4** from C-1 to C-37. The product ion of compound **4** at *m/z* 903, generated by the cleavage of the C-C bond located at C-38 and C-39, was not observed in the MS^2^ spectrum of compound **3**, while the product ion at *m/z* 885 formed by the loss of H_2_O from *m/z* 903 in compound **4** was observed in compound **3**, indicating that dehydroxylation occurred on C-38. The compound was thus named 38-dehydroxy-12,13-dihydro-44-methylgambierone. The proposed attributions of the ion formulas, along with mass differences, are presented in Table S4. 

#### 2.2.3. Fragmentation Pathways of 38-Dehydroxy-44-Methylgambierone

**Figure 6:**
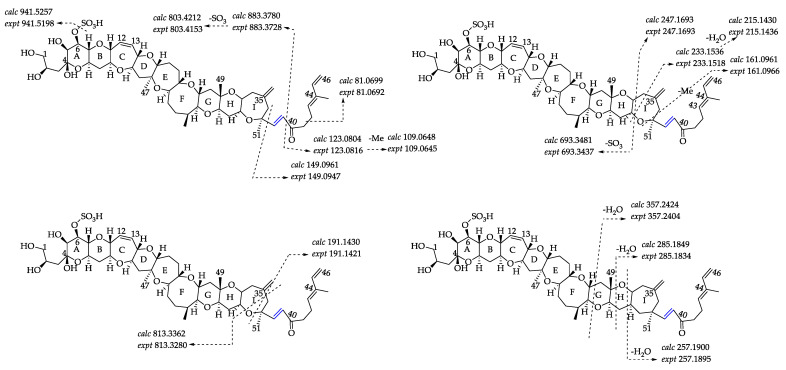

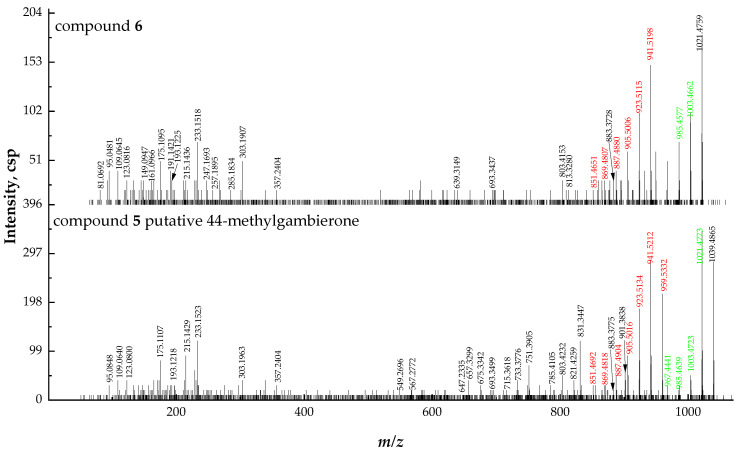
Fragment ion spectra (MS^2^, MS/MS) of [M + H]^+^ ions of compounds **6** and **5**, and fragmentation proposed for compound **6**. The product ions formed by H_2_O losses from precursor ions are shown in green, and the ions formed by the SO_3_ loss plus H_2_O losses from precursor ions are shown in red.

Compound **6** ([M + H]^+^ *m/z* 1021.4759, C_52_H_77_O_18_S^+^, Δ 6.5 ppm) was proposed to be 38-dehydroxy-44-methylgambierone, according to its similar features to those of 44-methylgambierone, including retention time, ion ratios, adduct formation, the observation of SO_3_ (80 Da) loss, specific fragments (*m/z* 109.0645, Δ 2.8 ppm; *m/z* 233.1518, Δ 7.7 ppm), and the fragmentation pattern of sequential H_2_O losses (Figure 6). The proposed precursor ion [M + H]^+^ at *m/z* 1021 and product ion [M − SO_3_]^+^ at *m/z* 941 produced by the first SO_3_ loss from the precursor ion were 18 Da lower than those of 44-methylgambierone, suggesting that a hydroxyl group was removed from this compound in comparison with 44-methylgambierone. The observation of fragment ions at *m/z* 123, 109, and 81 indicated that compound **6** had the same structure as 44-methylgambierone from C-40 to C-46. The produced ions at *m/z* 813 and 693 revealed that the hydroxyls on C-1, C-2, C-4, and C-5 remained unchanged in compound **6**. Then, we deduced that the position of dehydroxylation occurred on C-38. The product ion of 44-methylgambierone at *m/z* 901 generated by the cleavage of the C-C bond located at C-38 and C-39 was not observed in the MS^2^ spectrum of compound **6**, while the product ion at *m/z* 883 formed by the loss of H_2_O from *m/z* 901 was observed in compound **6**, supporting the hypothesis that dehydroxylation occurred on C-38. The compound was named 38-dehydroxy-44-methylgambierone. The proposed attributions of the ion formulas, along with mass differences, are displayed in Table S5.

#### 2.2.4. Fragmentation Pathways of Desulfo-Hydroxyl Gambierone

**Figure 7:**
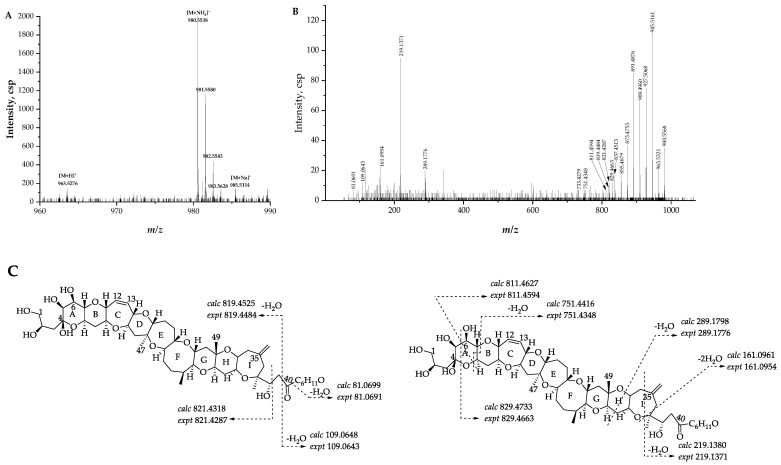
(**A**) Full-scan mass spectra (MS^1^) and (**B**) fragment ion spectra (MS^2^, MS/MS) of [M + NH_4_]^+^ ions of compound **2**; (**C**) fragmentation proposed for compound **2**.

Compound **2** ([M + NH_4_]^+^ *m/z* 980.5568, C_51_H_82_NO_17_^+^, Δ 1.5 ppm) has a similar structure to that of compound **1,** which is the putative gambierone. The full-scan mass spectrum of compound **2** showed putative proton, ammonium, and sodium adduct precursor ions at *m/z* 963, 980, and 985, respectively, and the ion ratio was similar to compound **1** (Figure 7A). Moreover, the MS^2^ spectrum of compound **2** showed similar product ions to compound **1** and other gambierone analogues (Figure 7B). The observation of the first neutral loss was inferred to be an ammonium loss (*m/z* 963.5321, Δ −0.9 ppm), and there were no fragments containing a sulfate group. The ammonium adduct precursor ion of compound **2** was 18 Da higher than the ammonium adduct ion at *m/z* 962 formed by SO_3_ loss from the precursor ion of compound **1**. In addition, the product ion at *m/z* 945, the first H_2_O loss proton adduct [M − H_2_O + H]^+^, was consistent with the loss of the SO_3_ proton adduct of gambierone [M − SO_3_ + H]^+^. The above information suggests that this gambierone analogue was desulfo-gambierone with an additional hydroxyl group. The product ion at *m/z* 821 was formed by the cleavage of the C-C bond between C-38 and C-39, corresponding to the fragment ion of 44-methylgambierone yielded by the breakage of the same site plus the loss of SO_3_, which indicated the same structure of the left moiety in compound **2**. The observation of fragment ions at *m/z* 219, 161, 109, and 81, possessing water loss, indicated that the additional hydroxyl group was located on one of the last six carbon atoms with no exact location determined. The compound was thus named as desulfo-hydroxyl gambierone. The fragmentation pathways and the proposed attributions of the ion formulas, along with mass differences, are presented in Figure 7C and Table S6, respectively.

### 2.3. Toxin Detection in Gambierdiscus balechii 1123M1M10

This study identified six gambierone analogues by comparing their MS^2^ spectra with standards and the reported MS data. Their fragmentation pathways were proposed with high accuracy between the monoisotopic mass of the measured ions and the theoretical ones. NMR analyses need to be conducted for further structural elucidation. Herein, gambierone and 44-methylgambierone were quantifiable in the algal crude extracts. The limits of detection (LOD) and quantification (LOQ) of analytical methods were determined by using the signal-to-noise (S/N) ratios of 3:1 and 10:1. The LODs of gambierone and 44-methylgambierone analyses were 0.09 ng/mL and 0.2 ng/mL, equating to 0.005 pg/cell and 0.01 pg/cell, and the LOQs were 0.3 ng/mL and 0.8 ng/mL, equating to 0.02 pg/cell and 0.05 pg/cell. The production level of gambierone (0.006 pg/cell) was below its LOQ and 44-methylgambierone was 4.0 pg/cell. 44-Methylgambierone was the predominant gambierone analogue in *G. balechii* 1123M1M10, accounting for around 40% of the total 6 gambierones with the comparison of their peak areas. 12,13-Dihydro-44-methylgambierone was the second most predominant gambierone analogue, accounting for around 30%, while the proportions of desulfo-hydroxyl gambierone, 38-dehydroxy-44-methylgambierone, 38-dehydroxy-44-methylgambierone, and gambierone were around 9%, 7%, 7%, and 2%, respectively. To date, six types of polyether toxins, including CTXs, MTXs, gambierones, gambierol, gambietic acid and gambieroxide, were found in different *Gambierdiscus* species, and it appears that not all *Gambierdiscus* species can produce CTXs, the most important CFP causative toxins (Table 1). Although *G. balechii* showed CTX-like activity based on the neuroblastoma cell (N2A) assay, no CTXs were detected via HPLC-HRMS analyses [46]. Gambierone, 44-methylgambierone, and other gambierone analogues were likely responsible for the CTX-like activity since these toxins showed weak activities on the sodium channel [8,41]. The bioactivities of gambierone analogues warrant further investigation. 

## 3. Materials and Methods

### 3.1. Culture of Gambierdiscus balechii 1123M1M10

In our previous report, the *G. balechii* strain 1123M1M10 was isolated from Marakei Island, Republic of Kiribati, located in the central Pacific Ocean [19]. The strain was cultured in f/2-Si medium prepared with artificial seawater with a salinity of 30 in the State Key Laboratory of Marine Pollution, the City University of Hong Kong, at 22 ± 1 ℃ under a 12h:12h (light/dark) cycle with a light intensity of 70–90 mol photon m^−2^s^−1^. 

### 3.2. Sample Preparation for HPLC-MS/MS Analysis

Algal cells (6.5 × 10^5^ cells) were collected by filtration with a 47 mm isopore PC membrane (10 μm pore size, Merck Millipore). The cell pellet was transferred to glass tubes and resuspended in 20 mL methanol (Merck, Darmstadt, Germany). Cells lysis was performed using an ultrasonic processor (Sonicator Q700, QSONICA, CT, USA) operated at 30 amplitudes for 2 min in pulse mode (5 s on, 5 s pause). The lysate was dried by evaporation under a gentle stream of high-purity nitrogen. The algal crude extracts underwent liquid–liquid partition using dichloromethane and 60% aqueous methanol three times to afford two fractions. The two fractions were dried and then redissolved in 4 mL methanol. Each fraction was diluted ten times for preliminary HPLC-QTOF-HRMS and HPLC-QTRAP-HRMS analyses. Then, the two fractions were combined and dissolved in 2 mL methanol and underwent separation using a Sephadex LH-20 column (1.5 cm × 60 cm). Then, they were eluted with methanol to afford 18 fractions for HPLC- QTOF-HRMS analysis. The procedure of elution was set up according to the observation of the colored bands. Fractions one and two comprised 40 mL and 10 mL and were collected at a flow rate of 0.3 mL/min. The fractions 3 to 5, 12, 16, and 17 comprised 10 mL and were collected at a flow rate of 0.8 mL/min. The fractions 6 to 11 and 13–15 comprised 5 mL and were collected at a flow rate of 0.8 mL/min. The last fraction, 18, comprised 100 mL and was collected at a flow rate of 0.8 mL/min. All fractions were dried via evaporation under a gentle stream of high-purity nitrogen and redissolved in 50–200 μL 50%–100% methanol. Gambierone and 44-methylgambierone standards were purchased from Laboratorio CIFGA S.A. (Lugo, Spain).

### 3.3. Instrumental Analysis

#### 3.3.1. HPLC-QTOF-HRMS Analysis 

The non-target analysis of toxins was performed using HPLC-HRMS consisting of an Agilent 1290 UPLC system (Agilent, Palo Alto, CA, USA) and a Sciex X500R QTOF system (AB Sciex, Foster City, CA, USA) operating with the IDA method. A 10 μL aliquot was injected into a Phenomenex Kinetex C18 column (100 × 2.1 mm i.d., 1.7 µm). Gradient elution at a flow rate of 0.2 mL/min was performed using: (A) Mill-Q water containing 0.02% formic acid (Merck, Darmstadt, Germany) and 2 mM ammonium acetate (Sigma Aldrich, MO, USA), and (B) 95% acetonitrile containing 0.02% formic acid and 2 mM ammonium acetate for the IDA method with a positive ESI mode; (A) Mill-Q water containing 2 mM ammonium acetate, and (B) 95% acetonitrile containing 2 mM ammonium acetate for the IDA method with a negative ESI mode. The gradient elution procedure was performed as follows: the initial gradient condition of the metabolite separation started at 30% B and increased to 60% B within 5 min. Then, it increased to 90% B within 30min and to 100% B within 40 min, and was kept for 5 min before returning to 30% B for 1 min. The column was equilibrated at initial gradient conditions for 4 min before the next injection. 

IDA methods were performed as follows: mass spectrometry was conducted in IDA mode with a TOF-MS full-scan analysis (0.25 s) and up to 10 dependent MS/MS analyses (0.1 s for each MS/MS analysis) per cycle. The TOF-MS full scan was operated with the mass range of *m/z* from 100 to 2000, and the dependent MS/MS was operated with the mass range of *m/z* from 50 to 2000 under the high-resolution mode. Two ions at *m/z* 132.90490 and 829.53933 were used for full mass calibration and their resolutions were above 21,000 and 29,000. Four ions at *m/z* 185.12845, 298.21252, 494.33370, and 607.41776 were used for MS^2^ calibration and their resolutions were above 21,000, 22,000, 25,000, and 26,000, respectively. Dynamic background subtraction was applied in the IDA criteria for dynamic exclusion. The fragment ions were generated from collision-induced dissociation with nitrogen under standardized collision energy (CE) = 35 V with collision energy spread (CES) = 15 V for the positive ESI mode, and CE = −35 V with CES = 0 V for the negative ESI mode. The other experimental parameters included: nebulizer gas (gas 1), 30 psi; heater gas (gas 2), 40 psi; curtain gas, 25 psi; ion source temperature, 500 °C; ion spray voltage floating, 5500 V for positive ESI mode and −4500 V for negative ESI mode; declustering potential, 80 V for positive ESI mode and −80 V for negative ESI mode; and full MS collision energy, 10 V for positive ESI mode, and −10 V for negative ESI mode. The gas used was nitrogen. 

MRM^HR^ methods were performed in the positive ESI mode as follows: mass spectrometry was conducted in the MRM^HR^ mode with a TOF-MS full scan analysis (0.1 s) and TOF MS/MS analyses (0.1 s for each selected precursor ion) per cycle. The TOF-MS full scan was operated with the mass range of *m/z* from 100 to 2000, and the MS/MS was operated with the mass range of *m/z* from 50 to 2000 under the high-resolution mode. The fragment ions were generated from collision-induced dissociation with nitrogen under standardized collision energy (CE) = 40 V with collision energy spread (CES) = 15 V. Other experimental parameters included: nebulizer gas (gas 1), 30 psi; heater gas (gas 2), 40 psi; curtain gas, 25 psi; ion source temperature, 500 °C; ion spray voltage floating, 5500 V; declustering potential, 80 V; and full MS collision energy, 10 V. The gas used was nitrogen. 

#### 3.3.2. HPLC-QTRAP-HRMS Analysis

The quantification of toxins was performed using HPLC-MS/MS consisting of an Agilent 1290 UPLC system (Agilent, Palo Alto, CA, USA) and a Sciex 5500 QTRAP mass spectrometer (Foster City, CA, USA) operating in a multiple reaction monitoring (MRM) negative ESI mode. A 5 μL aliquot was injected into a Phenomenex Kinetex C18 column (100 × 2.1 mm i.d., 1.7 µm). Gradient elution at a flow rate of 0.2 mL/min was performed using (A) Mill-Q water containing 0.02% formic acid and 2 mM ammonium acetate and (B) 95% acetonitrile containing 0.02% formic acid and 2 mM ammonium acetate. The gradient elution procedure was performed as follows: The initial gradient condition started at 30% B and increased to 100% B at 10 min. Then, it was kept for 0.9 min before returning to 30% B for 0.2 min, with a total run time of 12 min. Further details of the mass parameters of gambierone and 44-methylgambierone are given in Table S7.

## 4. Conclusions

Four new and two known gambierones were characterized from *G. balechii* 1123M1M10 via high-performance liquid chromatography coupled with electrospray ionization tandem mass spectrometry (HPLC/ESI-MS^2^) analysis, and their fragmentation behaviors and pathways were proposed. 44-Methylgambierone and 12,13-dihydro-44-methylgambierone were the major toxins in this strain. Further studies on the isolation and evaluating bioactivity of these gambierones need to be conducted to investigate their role in CFP.

## Figures and Tables

**Table 1:** List of toxins detected in *Gambierdiscus* via NMR or LC-MS/MS analyses.

Species	Toxins
*Gambierdiscus toxicus* [4,10,11,15,29,30,34,37,38]	P-CTX3C, P-CTX4A, P-CTX4B, 52-epi-54-deoxy-P-CTX1B, 54-deoxy-P-CTX1B, M-seco-P-CTX4A/B, 49-epi-P-CTX3C, 51-hydroxy-P-CTX3C, M-seco-P-CTX3C, M-seco-CTX3C methyl acetal, 44-methylgambierone, gambieric acid A, B, C and D, gambierol, gambieroxide, MTX-1, MTX-2
*Gambierdiscus polynesiensis* [31,32,33]	P-CTX3C, P-CTX3B, P-CTX4A, P-CTX4B, M-seco-P-CTX3C, gambierone, 44-methylgambierone
*Gambierdiscus australes* [35,39,48,49]	gambierone, 44-methylgambierone, gambieric acid C and D, gambieroxide, MTX-1, MTX-5
*Gambierdiscus cheloniae* [24,36]	gambierone, 44-methylgambierone, MTX-6
*Gambierdiscus honu* [24,36]	gambierone, 44-methylgambierone, MTX-7
*Gambierdiscus excentricus* [7,40]	sulfo-gambierone, dihydro-sulfo-gambierone, MTX-4
*Gambierdiscus belizeanus* [41]	gambierone, 44-methylgambierone
*Gambierdiscus sylvae* [42]	gambierone, 44-methylgambierone
*Gambierdiscus pacificus* [6,24]	gambierone, 44-methylgambierone
*Gambierdiscus caribaeus* [24]	gambierone, 44-methylgambierone
*Gambierdiscus holmesii* [24,43]	gambierone, 44-methylgambierone
*Gambierdiscus lapillus* [24,44]	gambierone, 44-methylgambierone
*Gambierdiscus lewisii* [24,43]	gambierone, 44-methylgambierone
*Gambierdiscus carpenteri* [26,44]	44-methylgambierone
*Gambierdiscus balechii* [26]	44-methylgambierone
*Gambierdiscus scabrosus*	No information
*Gambierdiscus carolinianus*	No information
*Gambierdiscus jejuensis*	No information

## Supplementary Materials

The following supporting information can be downloaded at: https://www.mdpi.com/article/10.3390/md21010003/s1. Figure S1: Original extraction ion chromatograms (XICs) of putative gambierone analogues in fraction three, and XICs of standards of gambierone (5.92 min) at 345 ng/mL and 44-methylgambierone (6.32 min) at 315 ng/mL using the IDA method in the positive ESI mode; Figure S2: Fragment ion spectra (MS^2^, MS/MS) of [M − H]^−^ ions of gambierone, 44-methylgambierone, compound 4, and 5 using the IDA method in the negative ESI mode; Figure S3: Full-scan mass spectra (MS^1^) and fragment ion spectra (MS^2^, MS/MS) of [M + H]^+^ ion of gambierone standard using the IDA method in the positive ESI mode; Figure S4: Full-scan mass spectra (MS^1^) and fragment ion spectra (MS^2^, MS/MS) of [M + NH_4_]^+^ ion of compound 1 using the IDA method in the positive ESI mode; Figure S5: Full-scan mass spectra (MS^1^) and fragment ion spectra (MS^2^, MS/MS) of [M + H]^+^ ion of 44-methylgambierone standard using the IDA method in the positive ESI mode; Figure S6: Full-scan mass spectra (MS^1^) and fragment ion spectra (MS^2^, MS/MS) of [M + H]^+^ ion of compound 5 using the IDA method in the positive ESI mode; Figure S7: Full-scan mass spectra (MS^1^) and fragment ion spectra (MS^2^, MS/MS) of [M + H]^+^ ion of compound 4 using the IDA method in the positive ESI mode; Figure S8: Full-scan mass spectra (MS^1^) and fragment ion spectra (MS^2^, MS/MS) of [M + H]^+^ ion of compound 3 using the IDA method in the positive ESI mode; Figure S9: Full-scan mass spectra (MS^1^) and fragment ion spectra (MS^2^, MS/MS) of [M + H]^+^ ion of compound 6 using the IDA method in the positive ESI mode; Figure S10: Full-scan mass spectra (MS^1^) and fragment ion spectra (MS^2^, MS/MS) of [M + H]^+^ ion of compound 2 using the IDA method in the positive ESI mode; Figure S11: Calibration curves of gambierone and 44-methylgambierone; Table S1: The proposed attributions of ion formulas along with mass differences (Δ ppm) of compound 1; Table S2: The proposed attributions of ion formulas along with mass differences (Δ ppm) of compound 5; Table S3: The proposed attributions of ion formulas along with mass differences (Δ ppm) of compound 4; Table S4: The proposed attributions of ion formulas along with mass differences (Δ ppm) of compound 3; Table S5: The proposed attributions of ion formulas along with mass differences (Δ ppm) of compound 6; Table S6: The proposed attributions of ion formulas along with mass differences (Δ ppm) of compound 2; Table S7: Mass spectrometer conditions for analysis of gambierone and 44-methylgambierone.
