# Supplementary material for: Characterization of New Gambierones Produced by Gambierdiscus balechii 1123M1M10
Source: Mar Drugs. 2022 Dec 21;21(1):3. doi: 10.3390/md21010003 (PMC9866745; doi:10.3390/md21010003)
Supplement: Supplementary file 1 [file marinedrugs-21-00003-s001.zip › marinedrugs-2054873-supplementary.pdf]

marine drugs

MDPI

Supplementary Information

Article

Characterization of New Gambierones Produced by *Gambierdiscus balechii* 1123M1M10

Xiaowan Liu [1], Yihan Ma [2], Jiajun Wu [1,3], Qizhao Yin[2], Pengbin Wang [4,5], Jingyi Zhu [1], Leo Lai, Chan [1,3*] and Bin Wu [2*]

[1] The State Key Laboratory of Marine Pollution and Department of Biomedical Sciences, City University of Hong Kong, Hong Kong SAR 999077, China; xiaowliu5-c@my.cityu.edu.hk (X.L.); jiajunwu@cityu.edu.hk (J.W.); jingyizhu9-c@my.cityu.edu.hk (J.Z.)
[2] Ocean College, Zhejiang University, Zhoushan 321000, China; mayihan@zju.edu.cn (Y.M.); qizhaoyin@zju.edu.cn (Q.Y.)
[3] Shenzhen Key Laboratory for the Sustainable Use of Marine Biodiversity, Research Centre for the Oceans and Human Health, City University of Hong Kong Shenzhen Research Institute, Shenzhen 518057, China
[4] Key Laboratory of Marine Ecosystem Dynamics, Second Institute of Oceanography, Ministry of Natural Resources, Hangzhou 310012, China; algae@sio.org.cn (P.W.)
[5] The Fourth Institute of Oceanography, Ministry of Natural Resources, Beihai 536000, China
* Correspondence: wubin@zju.edu.cn (B.W.); leochan@cityu.edu.hk (L.L.C.)

Contents:

Figure S1. Original extraction ion chromatograms (XICs) of putative gambierone analogues in fraction three, and XICs of standards of gambierone (5.92 min) at 345 ng/mL and 44-methylgambierone (6.32 min) at 315 ng/mL using the IDA method in the positive ESI mode. 3

Figure S2. Fragment ion spectra (MS2, MS/MS) of [M-H] - ions of gambierone, 44-methylgambierone, compound 4, and 5 using the IDA method in the negative ESI mode. 5

Figure S3. Full-scan mass spectra (MS1) and fragment ion spectra (MS2, MS/MS) of [M+H]+ ion of gambierone standard using the IDA method in the positive ESI mode.6

Figure S4. Full-scan mass spectra (MS1) and fragment ion spectra (MS2, MS/MS) of [M+NH4]+ ion of compound 1 using the IDA method in the positive ESI mode. 7

Figure S5. Full-scan mass spectra (MS1) and fragment ion spectra (MS2, MS/MS) of [M+H]+ ion of 44-methylgambierone standard using the IDA method in the positive ESI mode. 9

Figure S6. Full-scan mass spectra (MS1) and fragment ion spectra (MS2, MS/MS) of [M+H]+ ion of compound 5 using the IDA method in the positive ESI mode. 11

Figure S7. Full-scan mass spectra (MS1) and fragment ion spectra (MS2, MS/MS) of [M+H]+ ion of compound 4 using the IDA method in the positive ESI mode. 13

Figure S8. Full-scan mass spectra (MS1) and fragment ion spectra (MS2, MS/MS) of [M+H]+ ion of compound 3 using the IDA method in the positive ESI mode. 15

Figure S9. Full-scan mass spectra (MS1) and fragment ion spectra (MS2, MS/MS) of [M+H]+ ion of compound 6 using the IDA method in the positive ESI mode. 16

Figure S10. Full-scan mass spectra (MS1) and fragment ion spectra (MS2, MS/MS) of [M+H]+ ion of compound 2 using the IDA method in the positive ESI mode. 17

Figure S11. Calibration curves of gambierone and 44-methylgambierone. 18

Table S1. The proposed attributions of ion formulas along with mass differences (Δ ppm) of compound 1 19

Table S2. The proposed attributions of ion formulas along with mass differences (Δ ppm) of compound 5 20

Table S3. The proposed attributions of ion formulas along with mass differences (Δ ppm) of compound 4 21

Table S4. The proposed attributions of ion formulas along with mass differences (Δ ppm) of compound 3 22

Table S5. The proposed attributions of ion formulas along with mass differences (Δ ppm) of compound 6 23

Table S6. The proposed attributions of ion formulas along with mass differences (Δ ppm) of compound 2 24

Table S7. Mass spectrometer conditions for analysis of gambierone and 44-methylgambierone 24

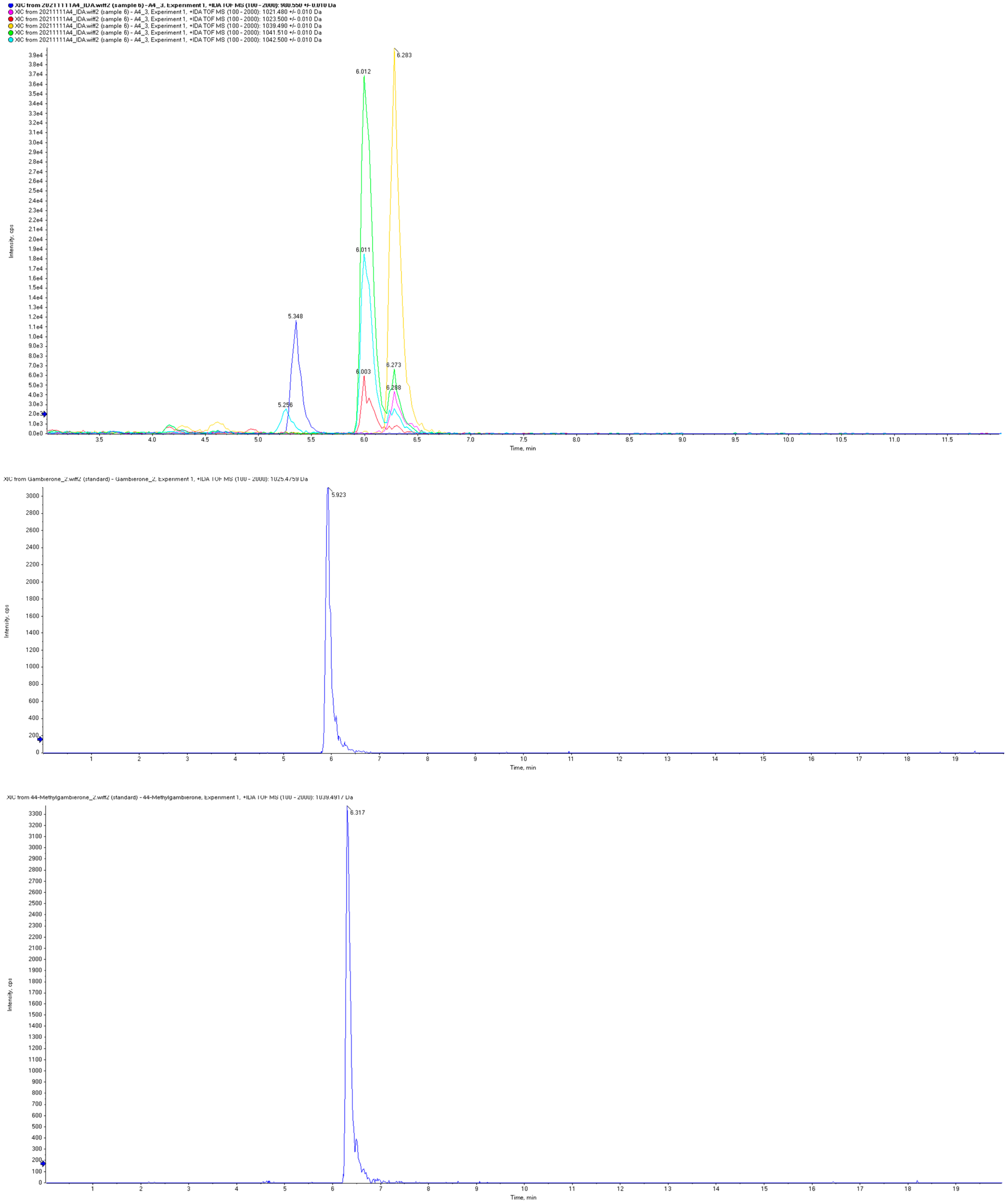

Figure S1. Original extraction ion chromatograms (XICs) of putative gambierone analogues in fraction three, and XICs of standards of gambierone (5.92 min) at 345 ng/mL and 44-methylgambierone (6.32 min) at 315 ng/mL using the IDA method in the positive ESI mode.

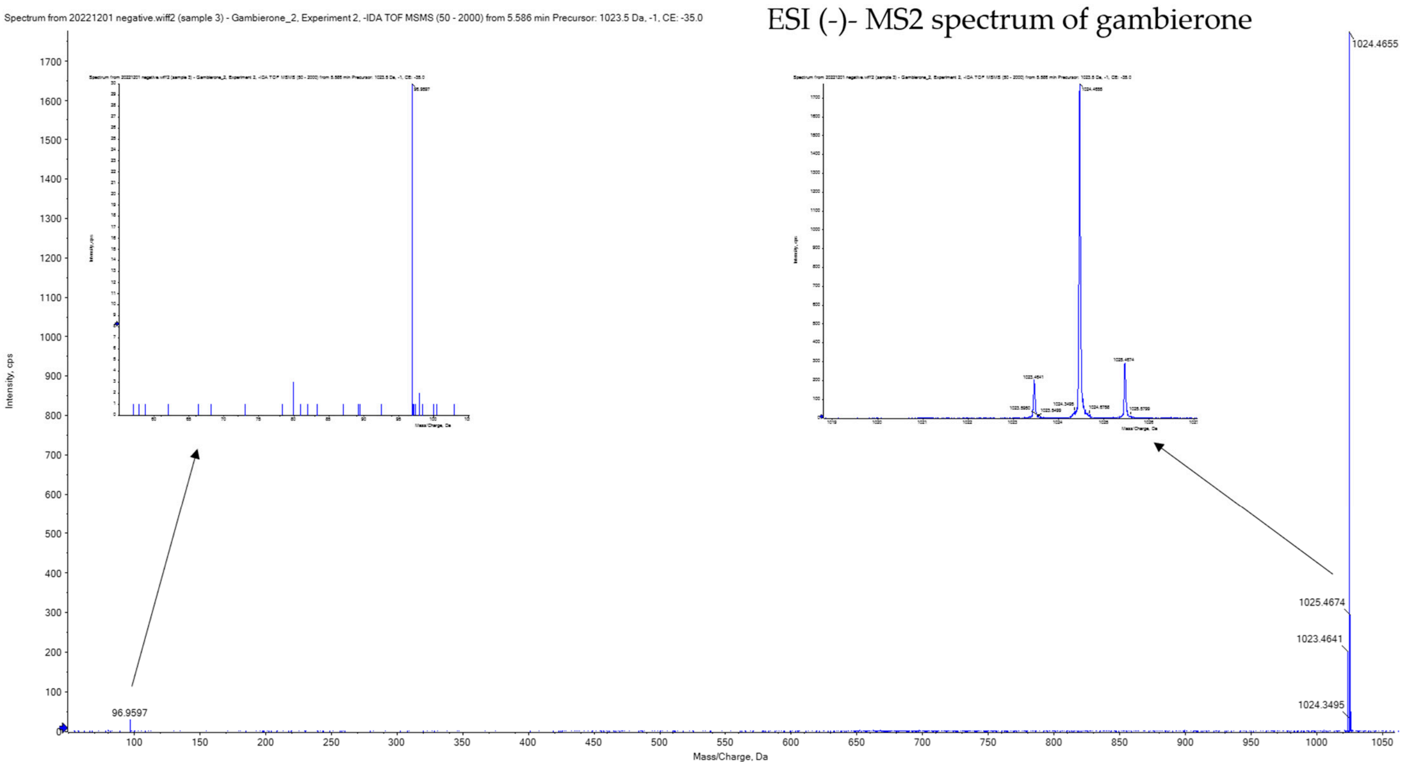
Spectrum from 20221201 negative.wiff2 (sample 3) - Gambierone_2, Experiment 2, -IDA TOF MSMS (50 - 2000) from 5.586 min Precursor: 1023.5 Da, -1, CE: -35.0
ESI (-)- MS2 spectrum of gambierone
Intensity, cps
Mass/Charge, Da
96.9597
1024.4655
1025.4674
1023.4641
1024.3495

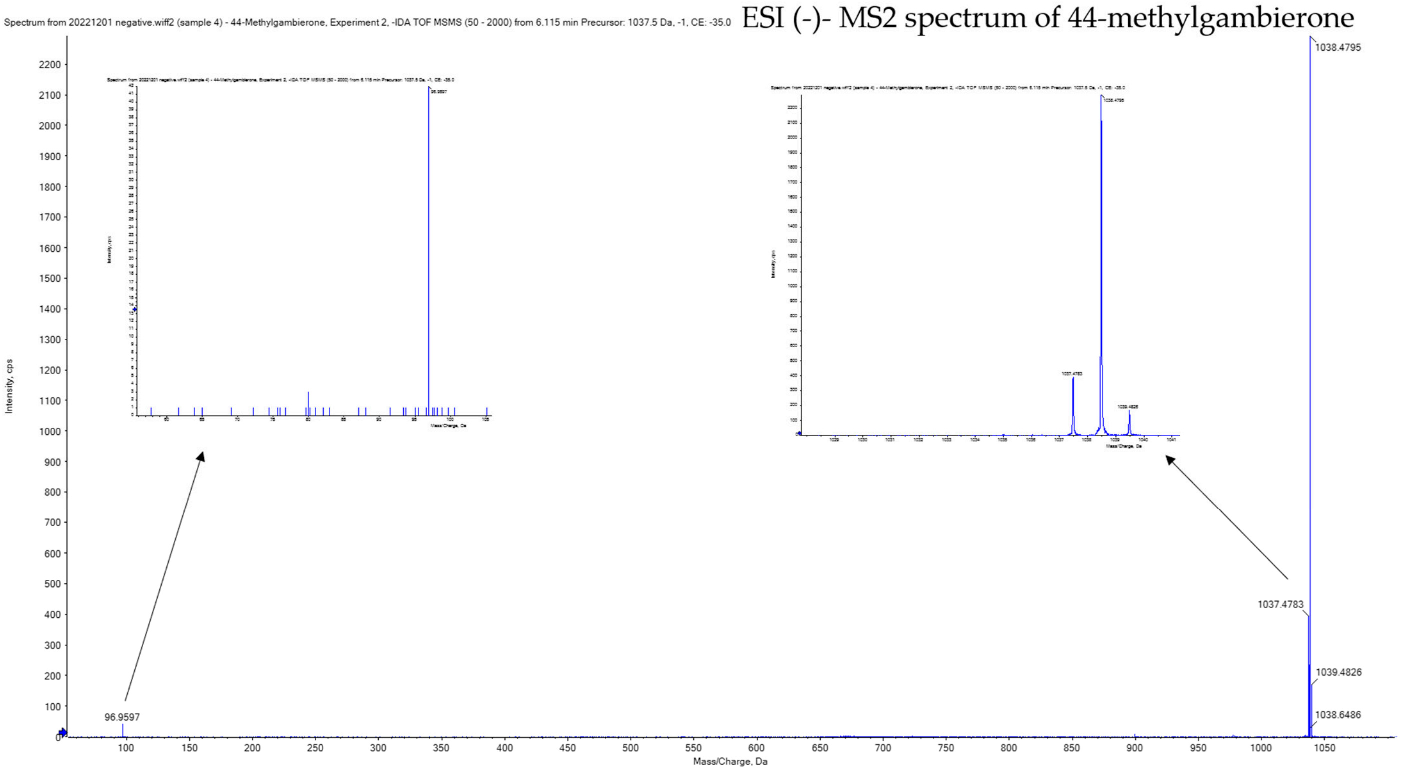
Spectrum from 20221201 negative.wiff2 (sample 4) - 44-Methylgambierone, Experiment 2, -IDA TOF MSMS (50 - 2000) from 6.115 min Precursor: 1037.5 Da, -1, CE: -35.0
ESI (-)- MS2 spectrum of 44-methylgambierone
Intensity, cps
Mass/Charge, Da
96.9597
1038.4795
1037.4783
1039.4826
1038.6486

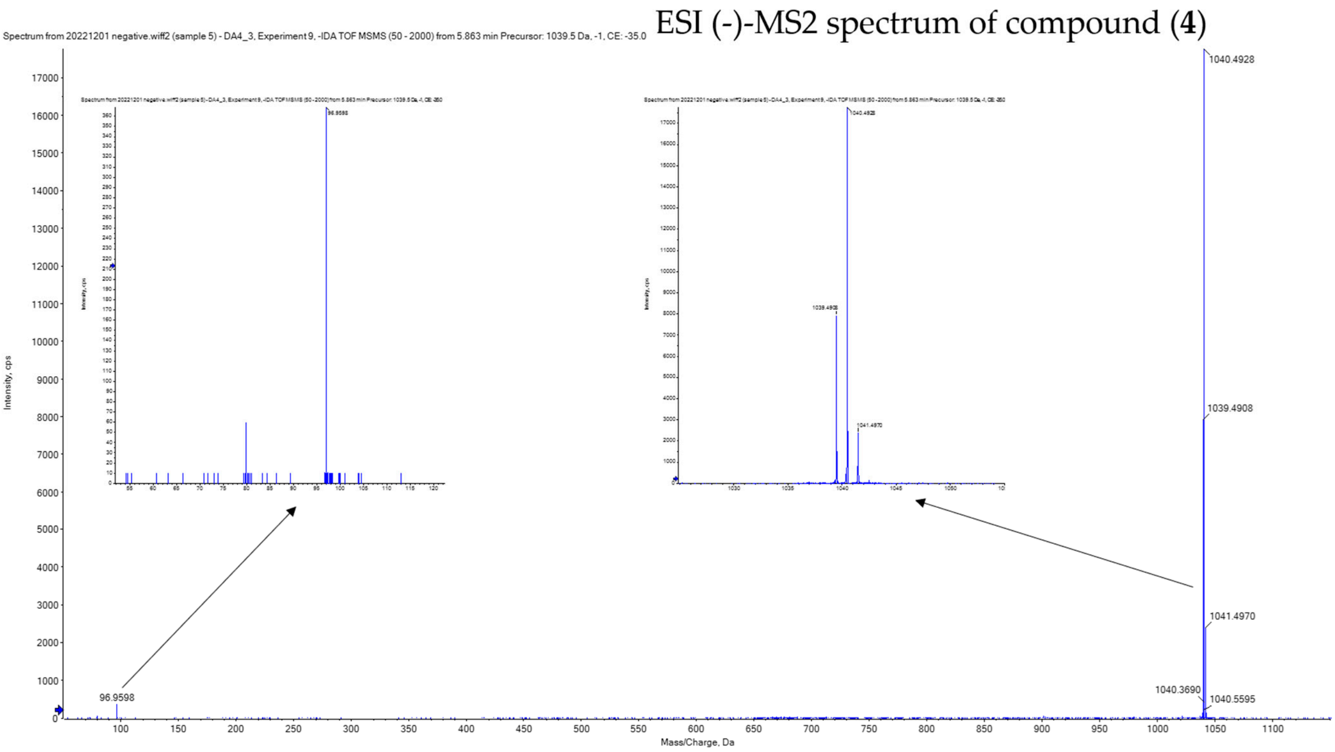

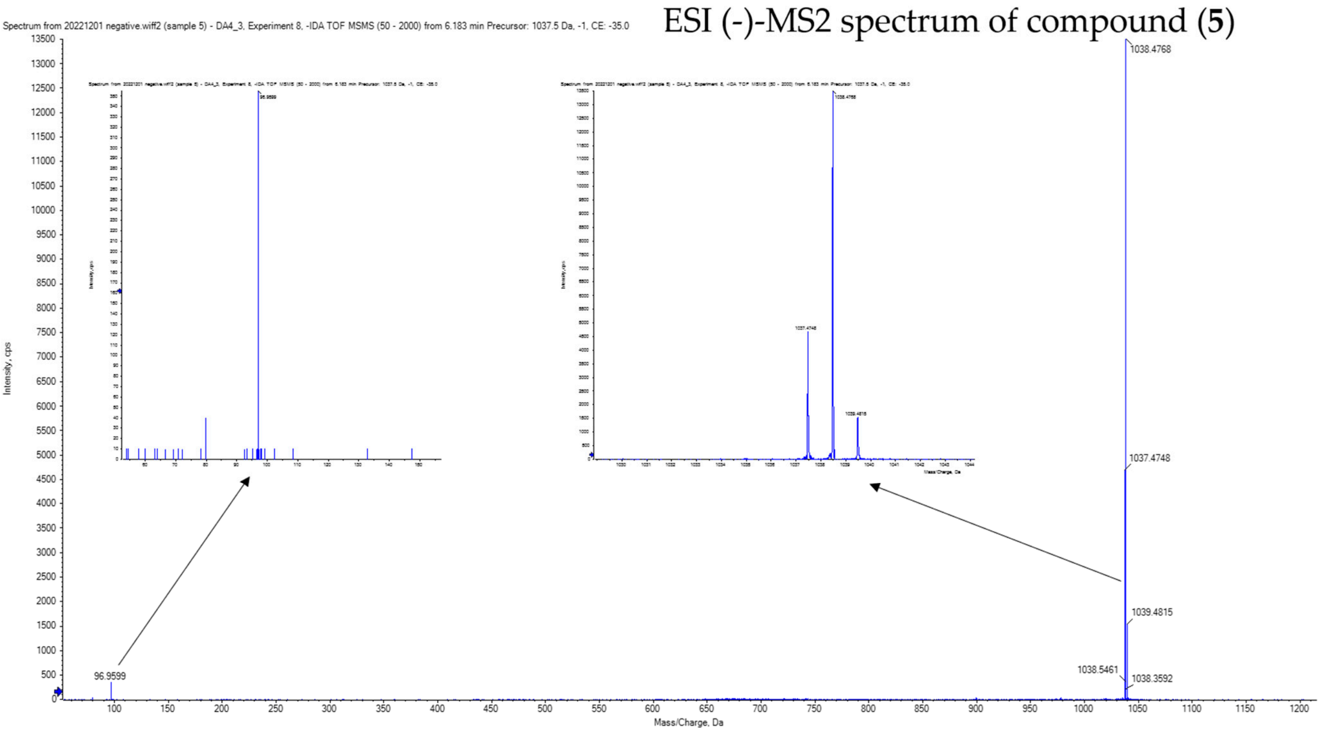

Figure S2. Fragment ion spectra (MS², MS/MS) of [M-H] ⁻ ions of gambierone, 44-methylgambierone, compound **4,** and **5** using the IDA method in the negative ESI mode.

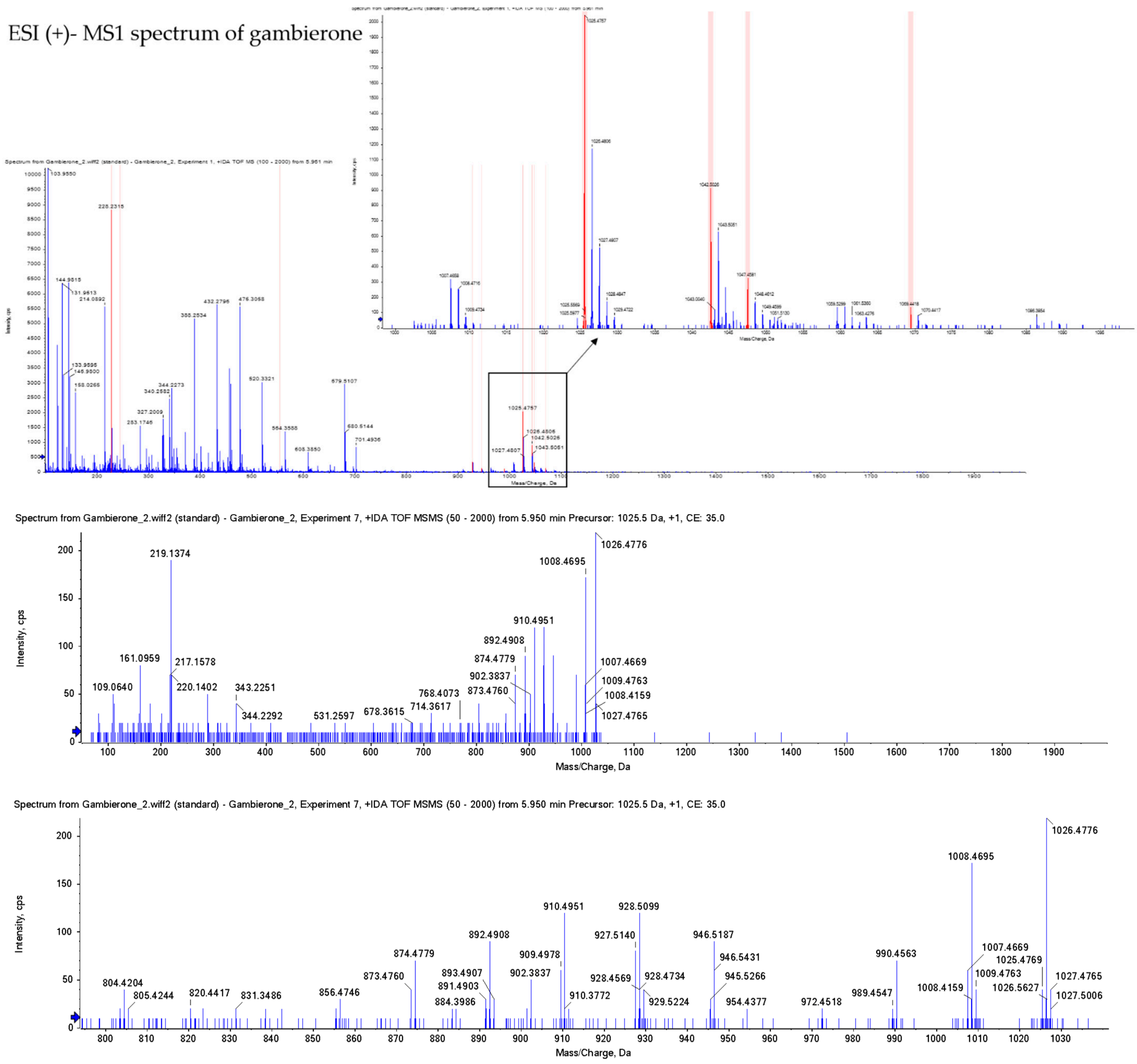

Figure S3. Full-scan mass spectra (MS¹) and fragment ion spectra (MS², MS/MS) of $[M+H]^+$ ion of gambierone standard using the IDA method in the positive ESI mode.

ESI (+)- MS1 spectrum of compound (**1**)

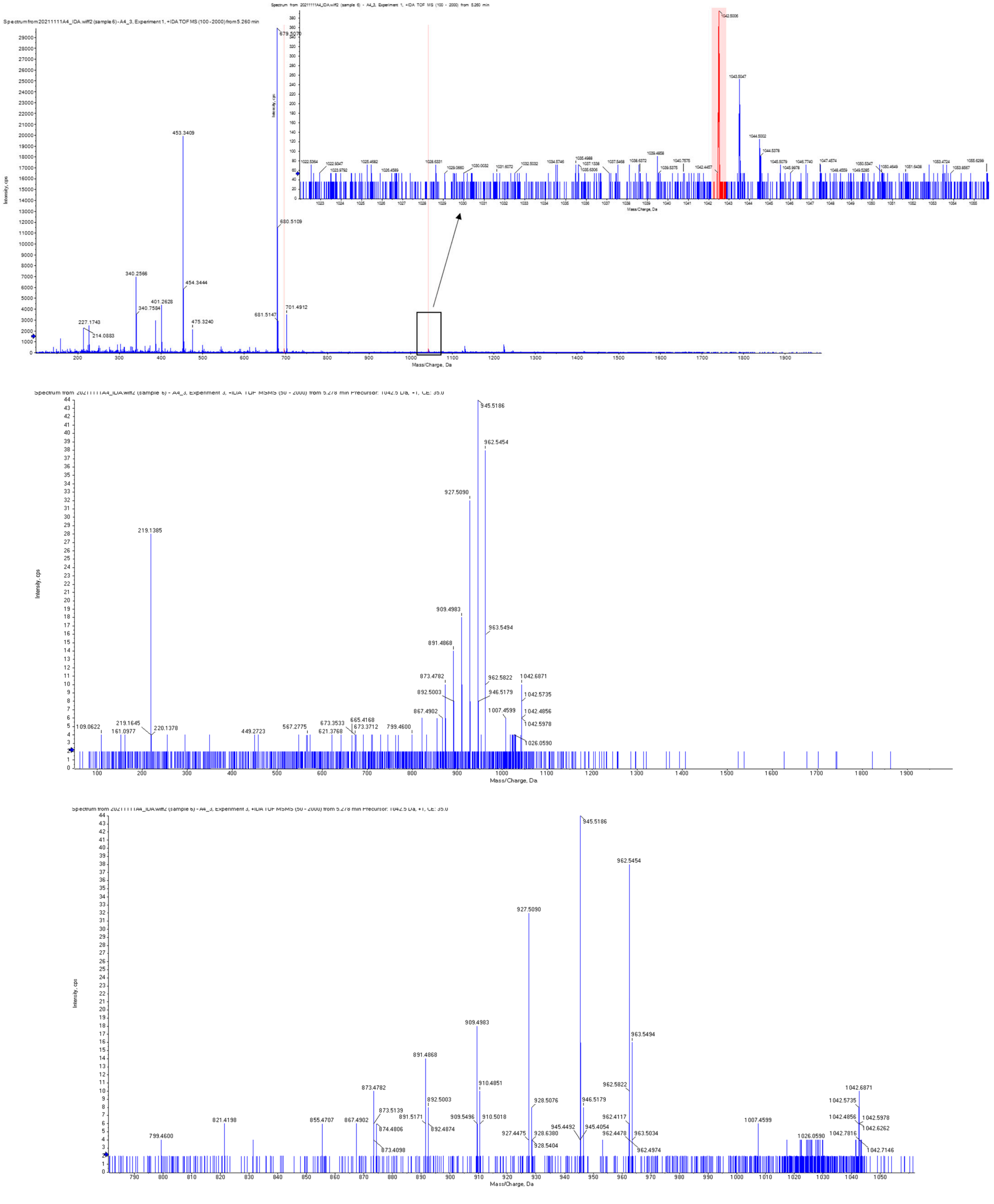

Figure S4. Full-scan mass spectra (MS^1) and fragment ion spectra (MS^2, MS/MS) of $[M+NH_4]^+$ ion of compound **1** using the IDA method in the positive ESI mode.

ESI (+)-MS1 spectrum of 44-methylgambierone standard

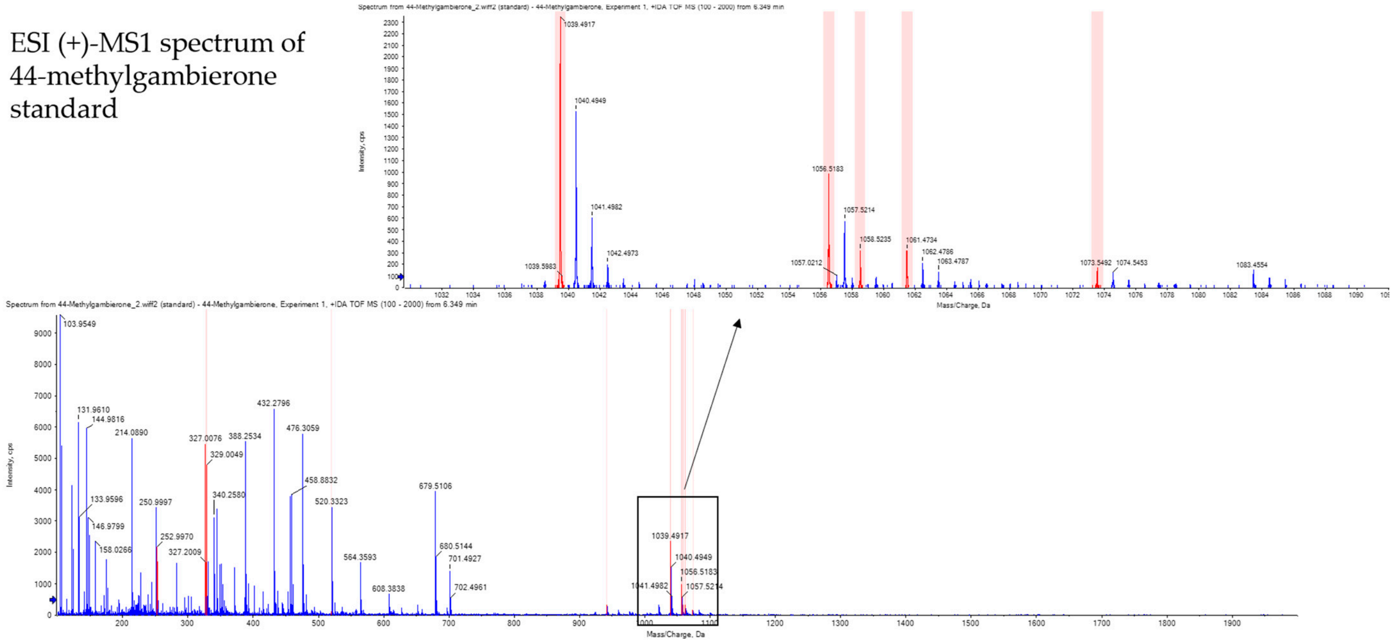

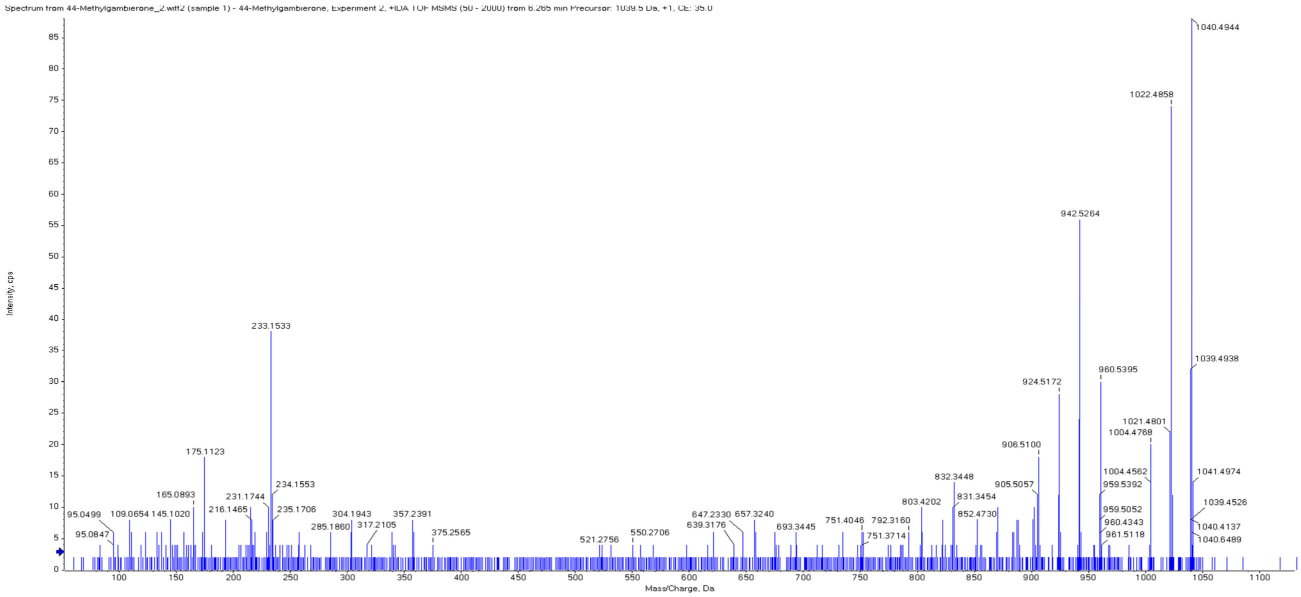

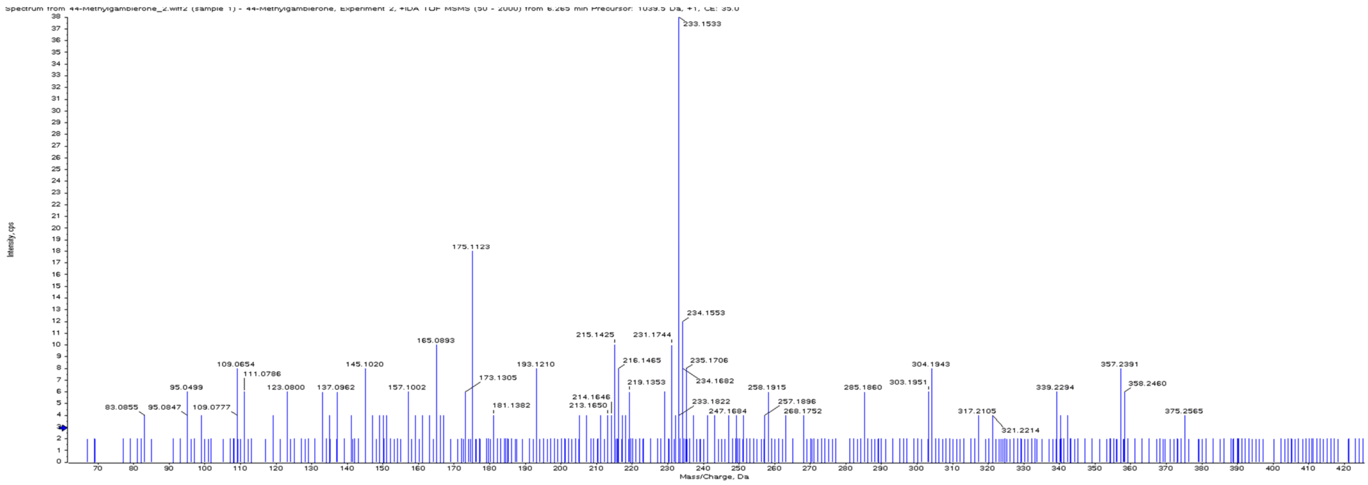

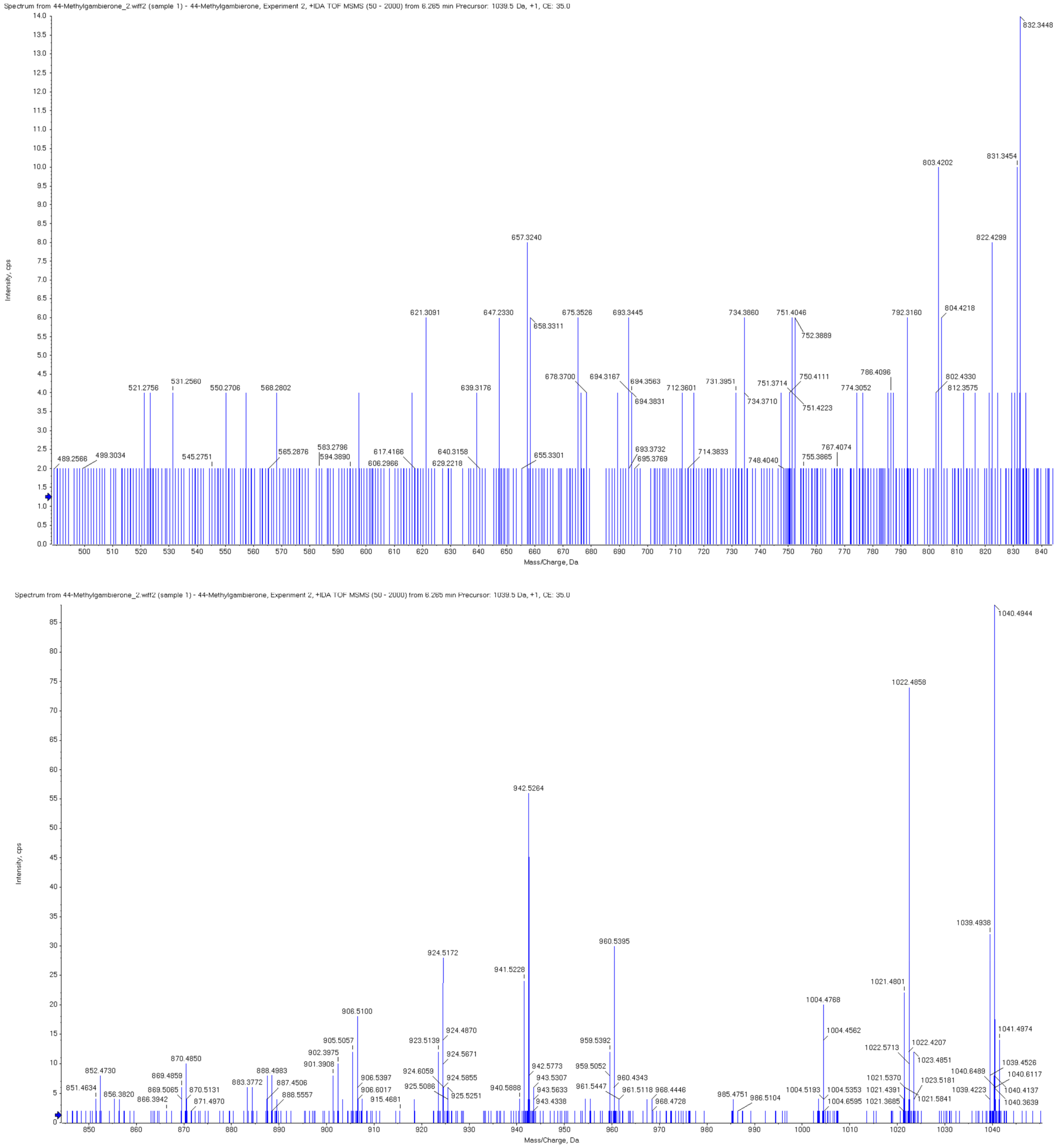

Figure S5. Full-scan mass spectra (MS¹) and fragment ion spectra (MS², MS/MS) of $[M+H]^+$ ion of 44-methylgambierone standard using the IDA method in the positive ESI mode.

ESI (+)-MS1 spectrum of compound (**5**)

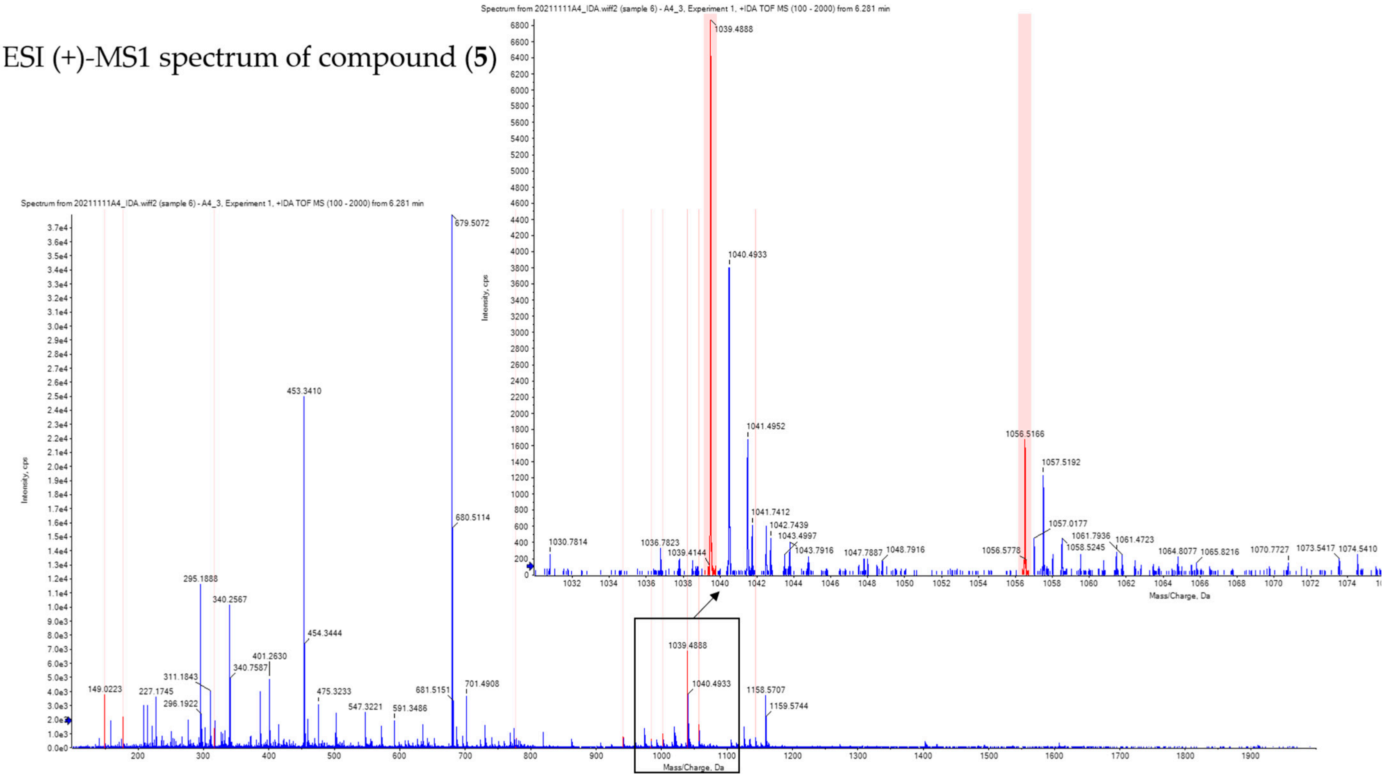

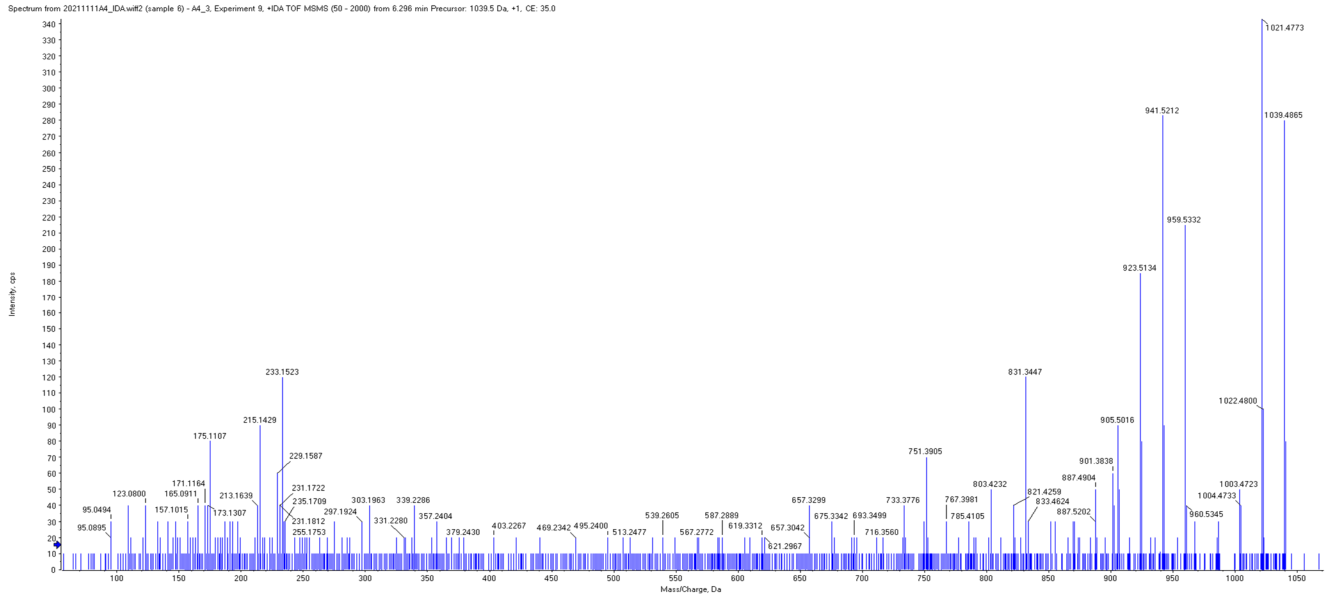

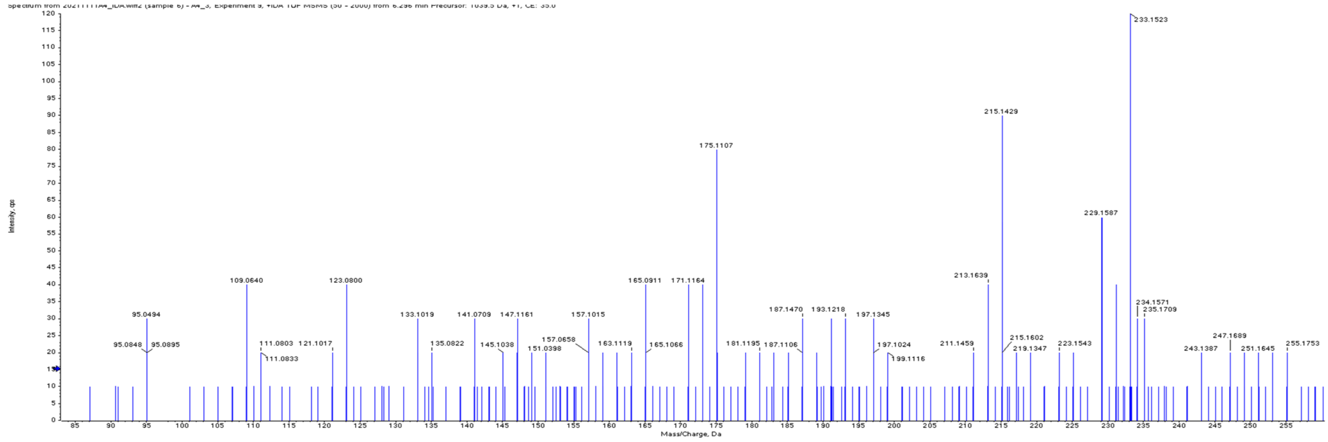

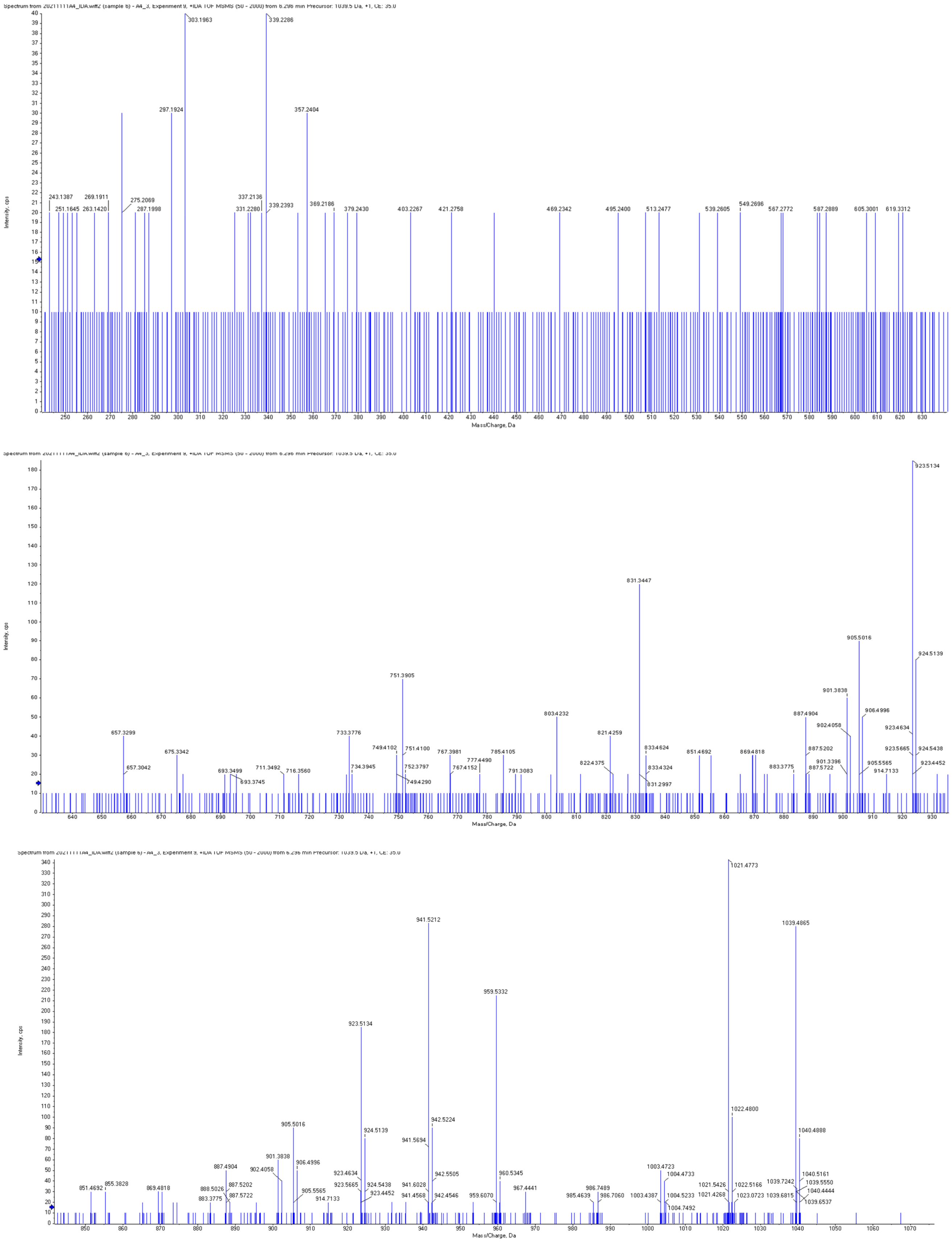

Figure S6. Full-scan mass spectra (MS^1) and fragment ion spectra (MS^2, MS/MS) of $[M+H]^+$ ion of compound **5** using the IDA method in the positive ESI mode.

ESI (+)-MS1 spectrum of compound (**4**)

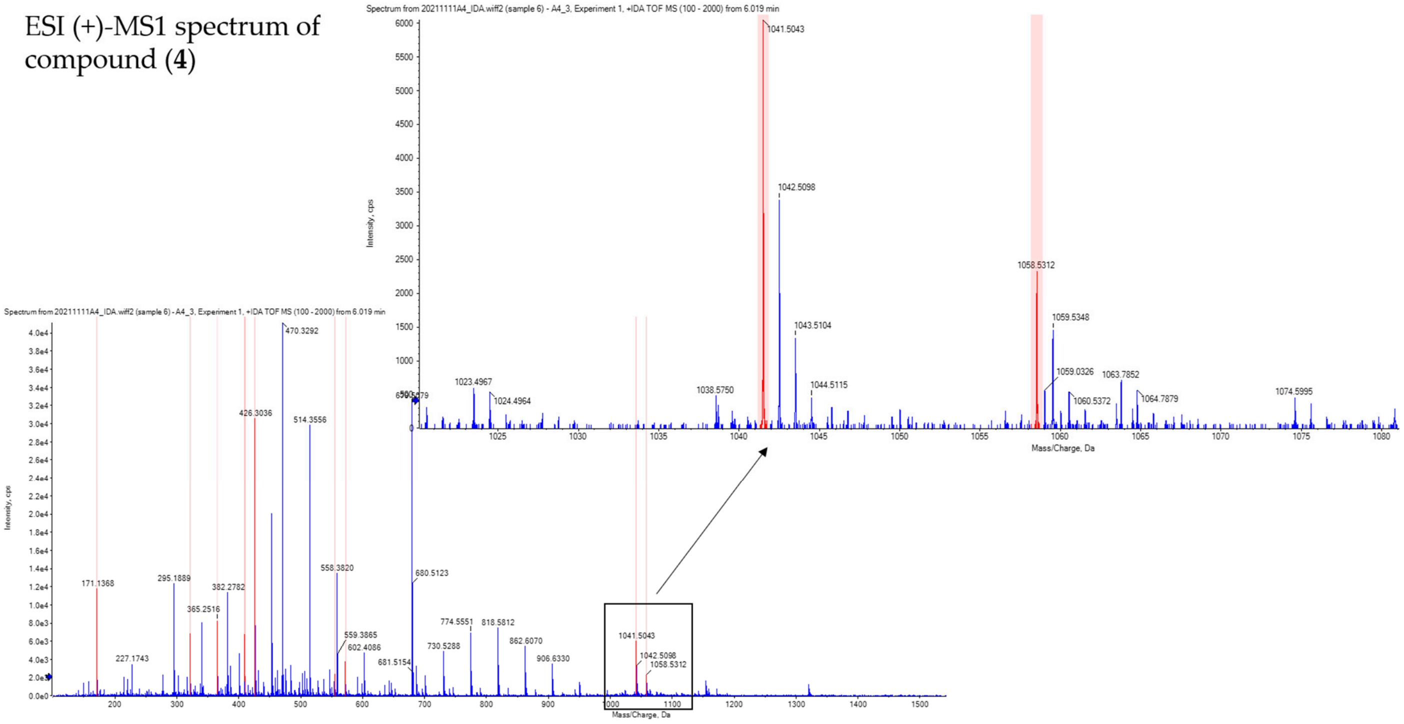

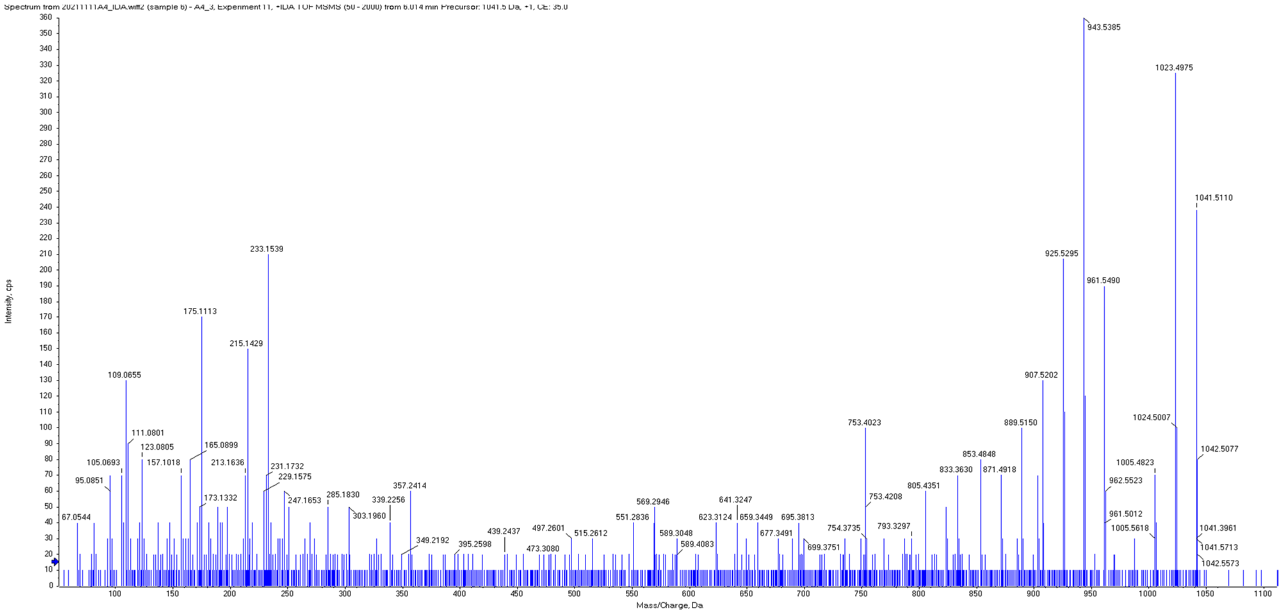

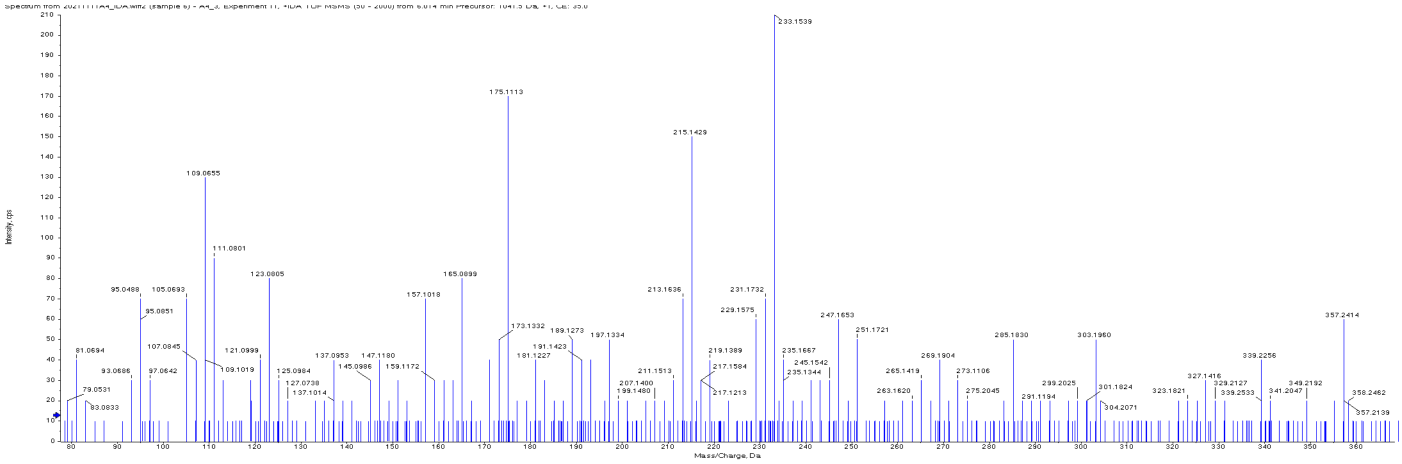

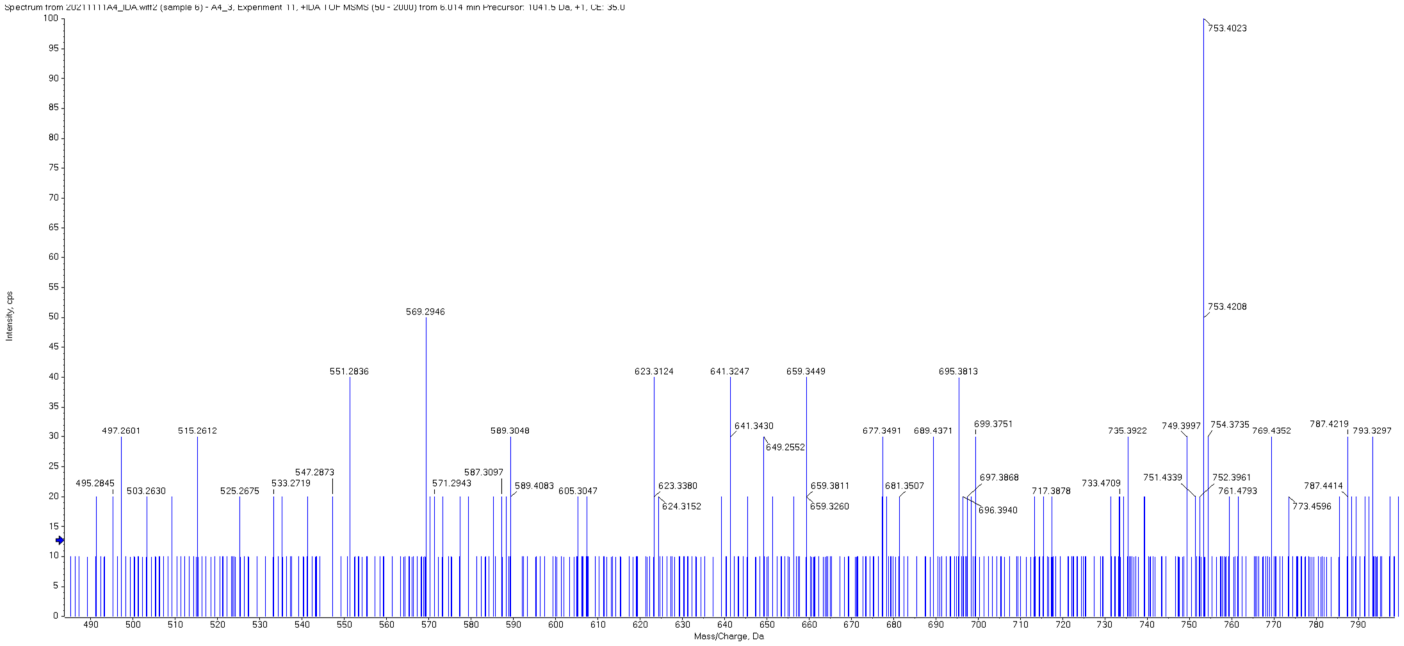

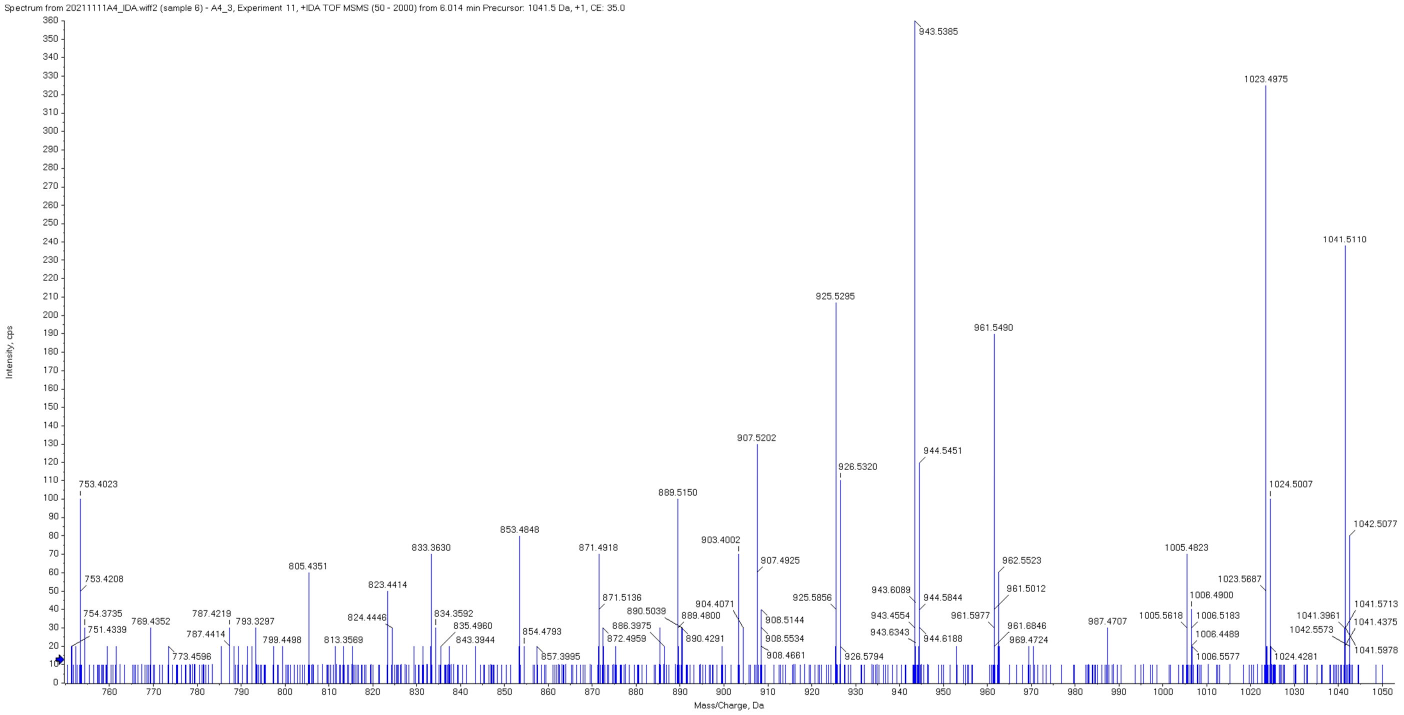

Figure S7. Full-scan mass spectra (MS^1) and fragment ion spectra (MS^2, MS/MS) of $[M+H]^+$ ion of compound **4** using the IDA method in the positive ESI mode.

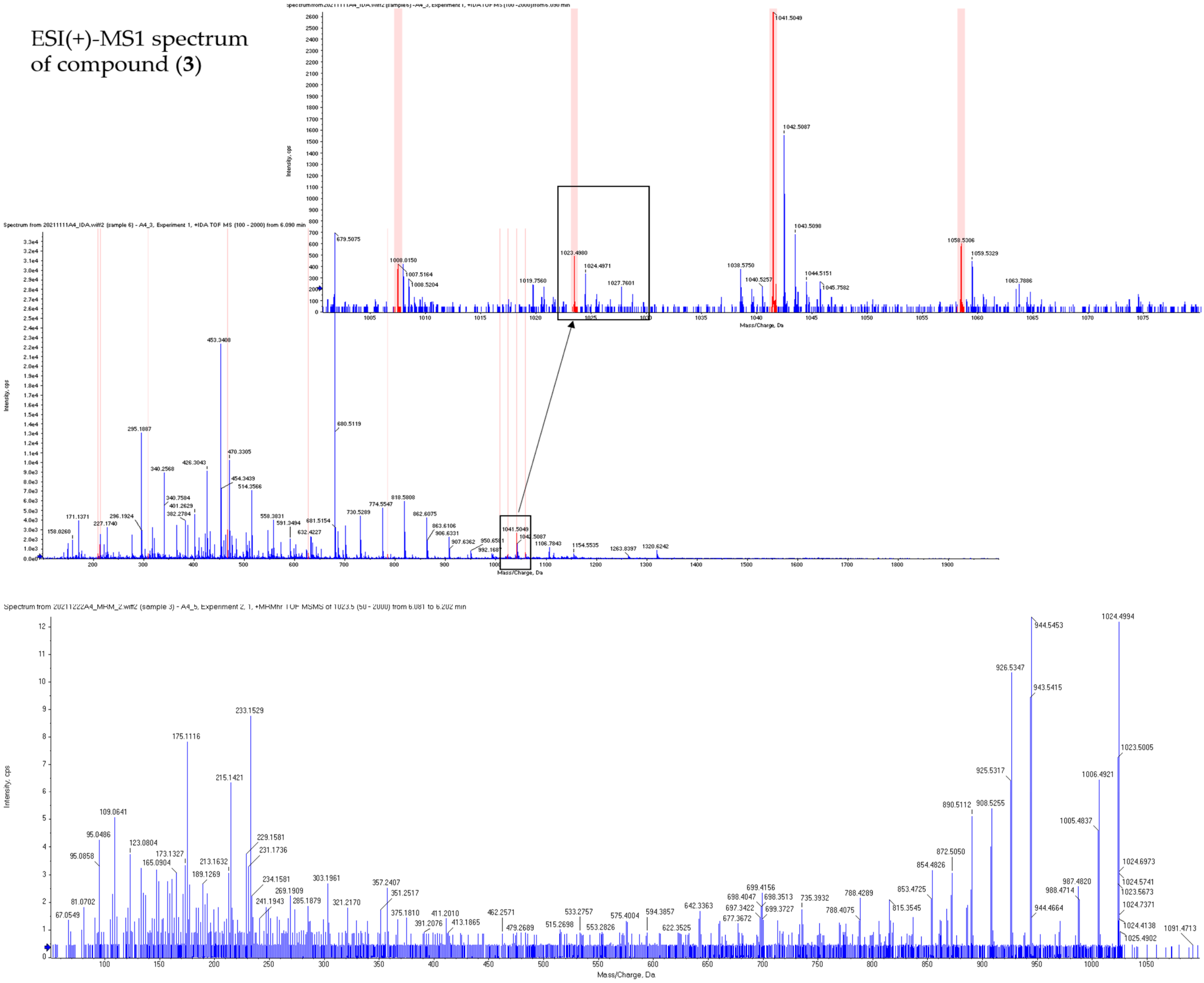

ESI(+)-MS1 spectrum of compound (**3**)
Intensity, cps
Mass/Charge, Da
1041.5049
1042.5087
1023.4980
1024.4971
453.3408
679.5075
680.5119
295.1887
470.3305
Spectrum from 20211222A4_MRM_2.wiff2 (sample 3) - A4_5, Experiment 2, 1, +MRMhr TOF MSMS of 1023.5 (50 - 2000) from 6.081 to 6.202 min
1024.4994
944.5453
926.5347
943.5415
1023.5005
233.1529
175.1116
215.1421
109.0641

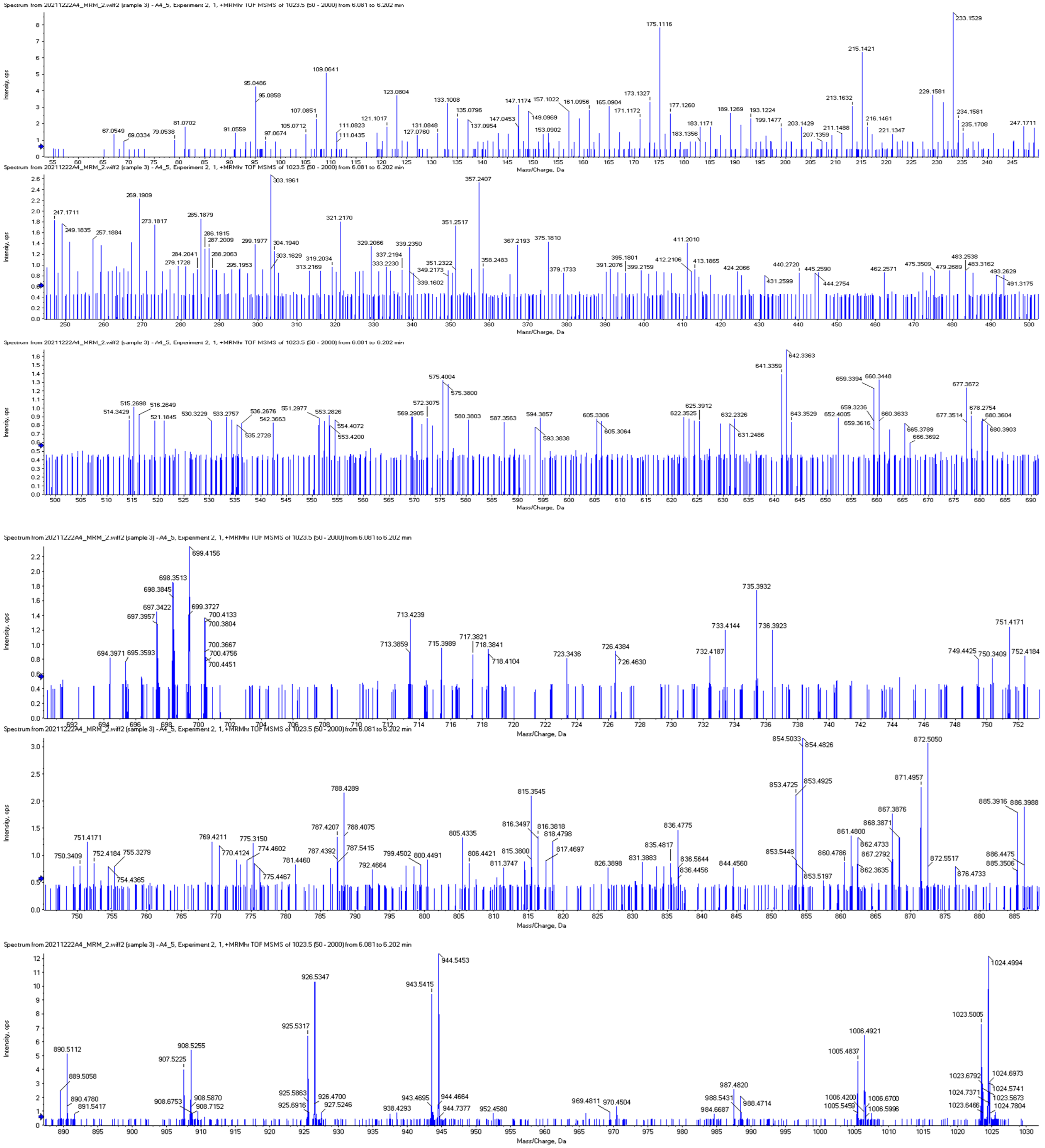

Figure S8. Full-scan mass spectra (MS[1]) and fragment ion spectra (MS[2], MS/MS) of [M+H]$^+$ ion of compound **3** using the IDA method in the positive ESI mode.

ESI(+)-MS1 spectrum of compound (**6**)

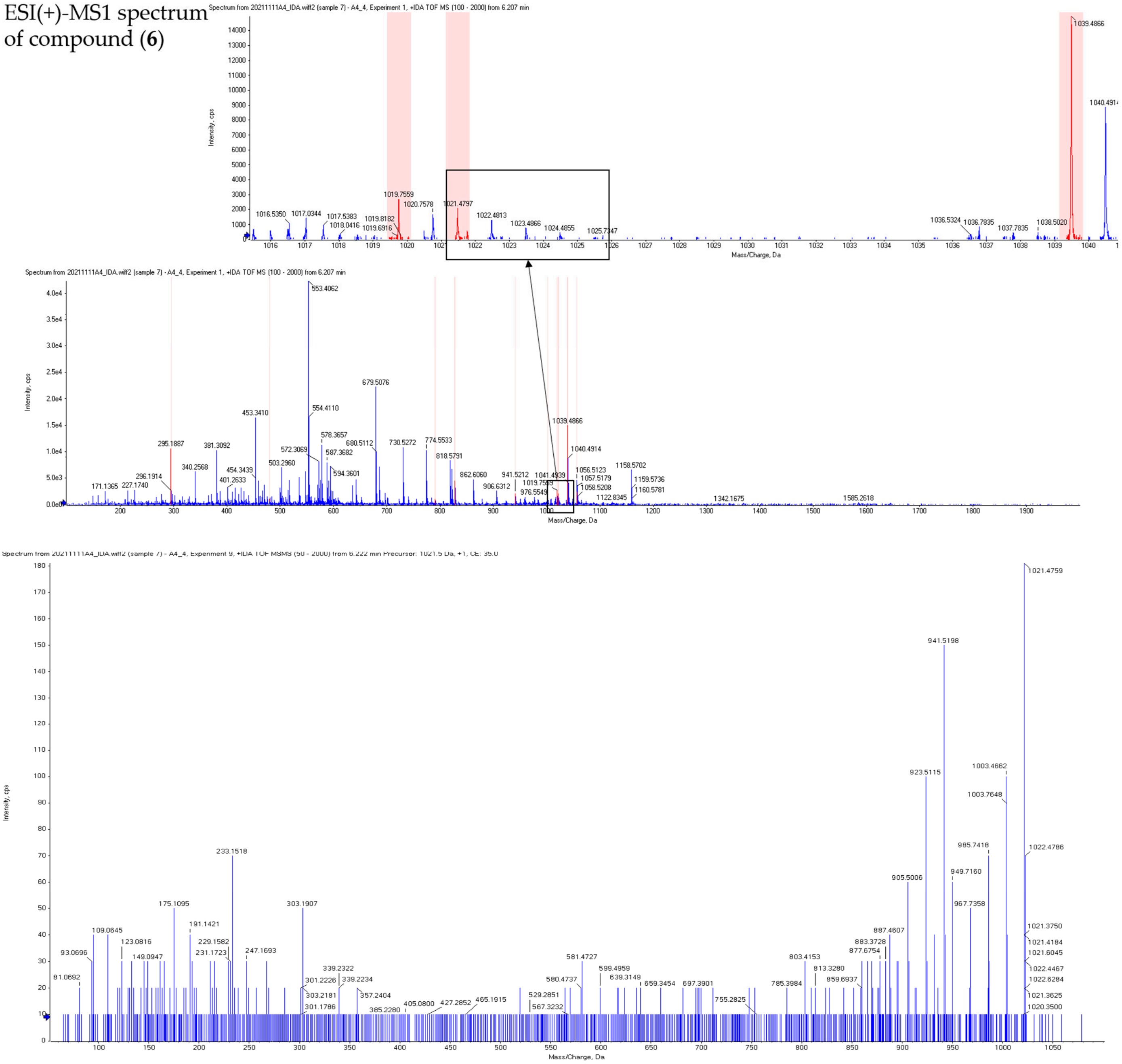

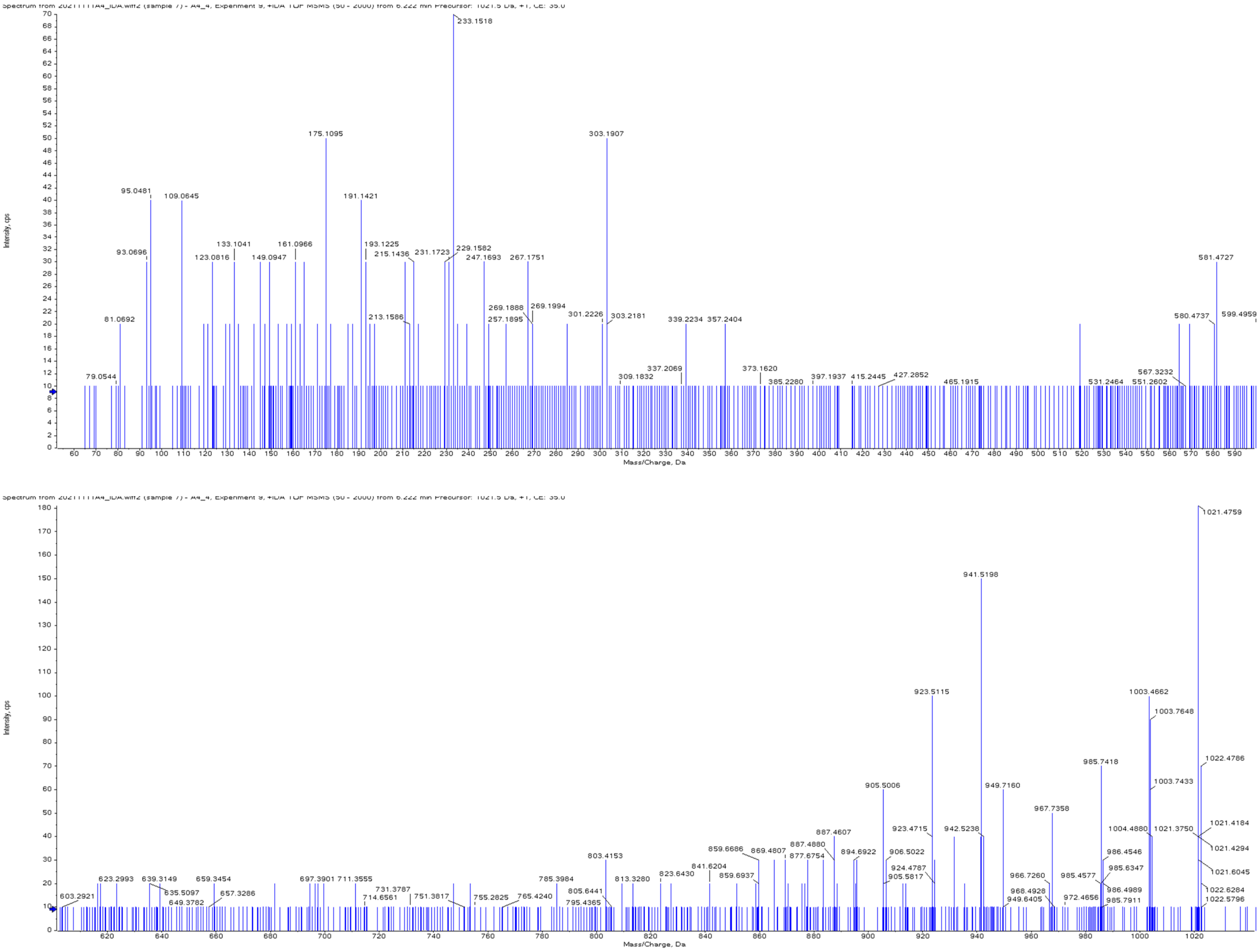

Figure S9. Full-scan mass spectra (MS[1]) and fragment ion spectra (MS[2], MS/MS) of $[M+H]^+$ ion of compound **6** using the IDA method in the positive ESI mode.

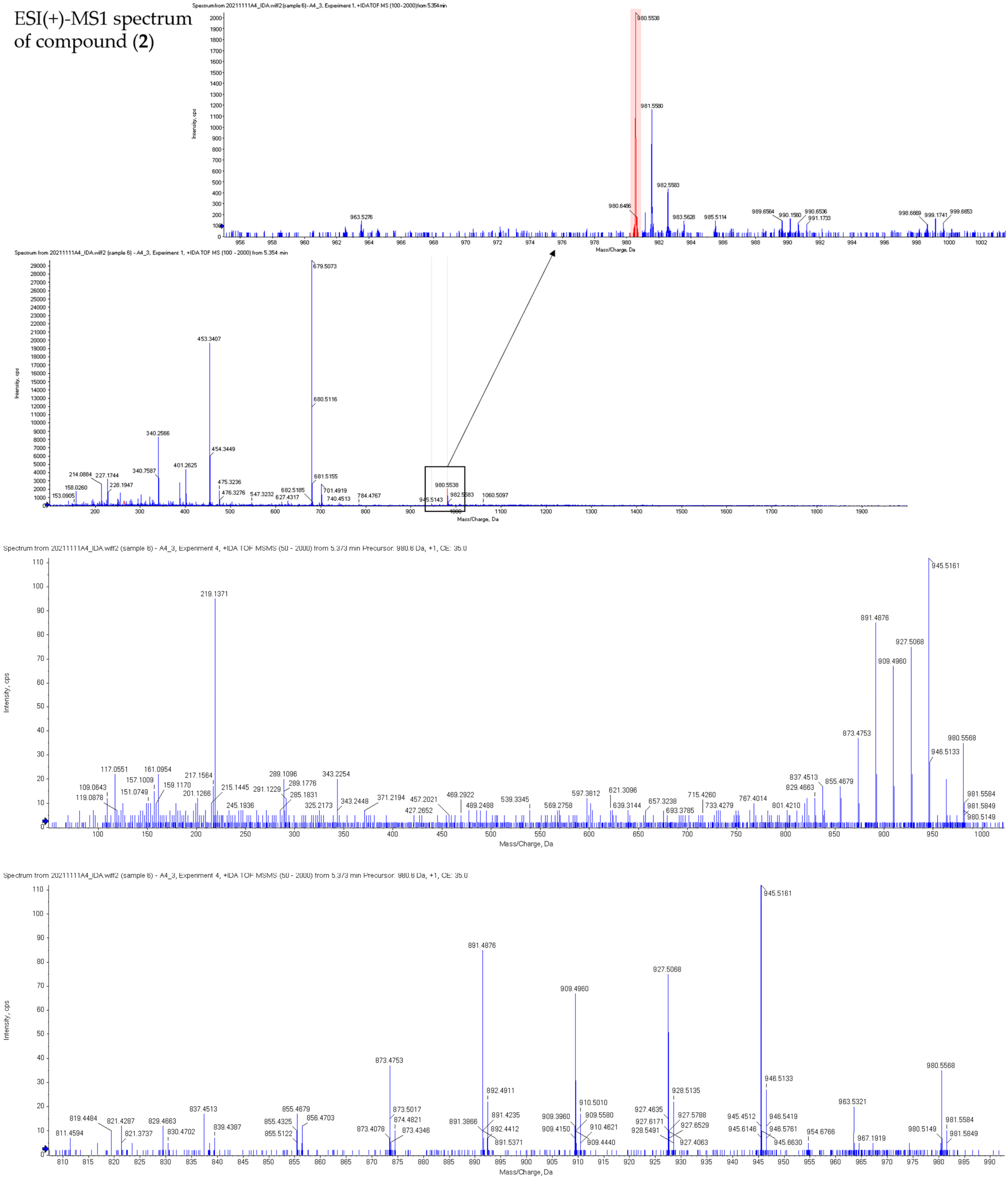

Figure S10. Full-scan mass spectra (MS[1]) and fragment ion spectra (MS[2], MS/MS) of $[M+H]^+$ ion of compound **2** using the IDA method in the positive ESI mode.

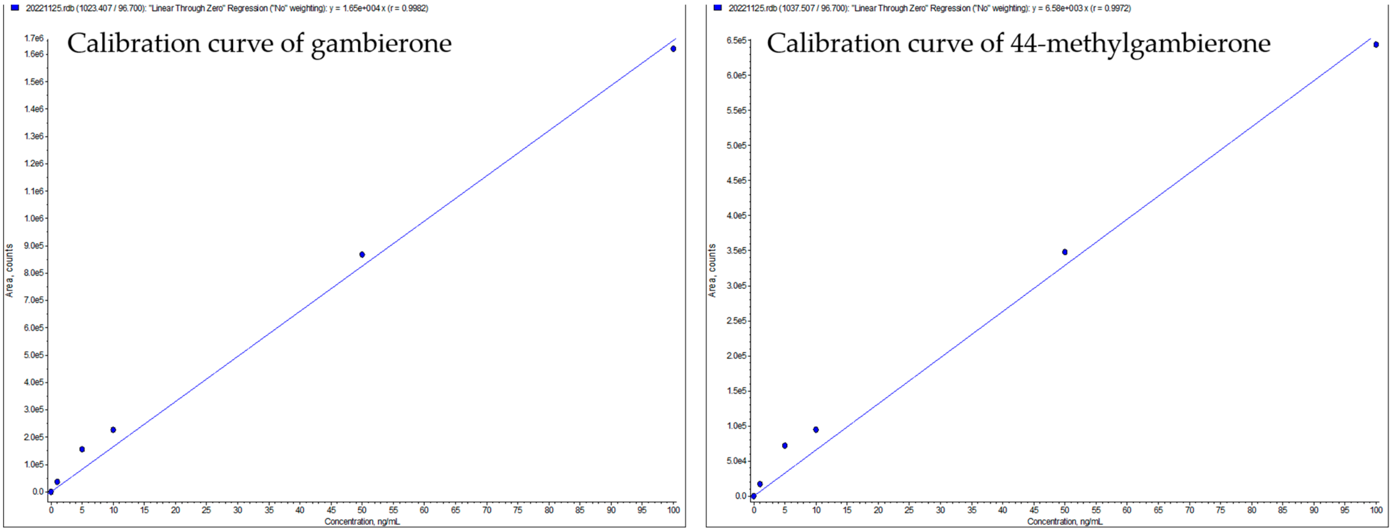

Figure S11. Calibration curves of gambierone and 44-methylgambierone.

Table S1. The proposed attributions of ion formulas along with mass differences (Δ ppm) of compound **1**

Elemental formula Ion	Theoretical *m*/*z*	Experimental *m*/*z*	Δ ppm	Elemental formula Ion	Theoretical *m*/*z*	Experimental *m*/*z*	Δ ppm
$C_{51}H_{80}NO_{19}S^+$	1042.5040	1042.5006	3.3	$C_{49}H_{71}O_{13}^+$	867.4889	867.4902	-1.5
$C_{51}H_{77}O_{19}S^+$	1025.4774	1025.4682	9.0	$C_{51}H_{67}O_{11}^+$	855.4678	855.4707	-3.4
$C_{51}H_{75}O_{18}S^+$	1007.4669	1007.4599	6.9	$C_{36}H_{55}O_{14}^+$	711.3586	711.3586	0
$C_{51}H_{80}NO_{16}^+$	962.5472	962.5454	1.9	$C_{38}H_{53}O_{7}^+$	621.3786	621.3768	2.9
$C_{51}H_{77}O_{16}^+$	945.5206	945.5186	2.1	$C_{29}H_{43}O_{11}^+$	567.2800	567.2775	4.4
$C_{51}H_{75}O_{15}^+$	927.5100	927.5090	1.1	$C_{28}H_{41}O_{5}^+$	457.2949	457.2927	4.8
$C_{51}H_{73}O_{14}^+$	909.4995	909.4983	1.3	$C_{14}H_{19}O_{2}^+$	219.1380	219.1385	-2.3
$C_{51}H_{71}O_{13}^+$	891.4889	891.4868	2.3	$C_{11}H_{13}O^+$	161.0961	161.0977	-9.9
$C_{51}H_{69}O_{12}^+$	873.4784	873.4782	0.2				

Table S2. The proposed attributions of ion formulas along with mass differences (Δ ppm) of compound **5**

Elemental formula Ion	Theoretical *m*/*z*	Experimental *m*/*z*	Δ ppm	Elemental formula Ion	Theoretical *m*/*z*	Experimental *m*/*z*	Δ ppm
$C_{52}H_{79}O_{19}S^+$	1039.4931	1039.4865	6.3	$C_{39}H_{57}O_{13}^+$	733.3794	733.3776	2.5
$C_{52}H_{77}O_{18}S^+$	1021.4825	1021.4773	5.1	$C_{39}H_{55}O_{12}^+$	715.3688	715.3618	9.8
$C_{52}H_{75}O_{17}S^+$	1003.4719	1003.4723	-0.4	$C_{36}H_{53}O_{13}^+$	693.3481	693.3499	-2.6
$C_{52}H_{73}O_{16}S^+$	985.4614	985.4639	-2.5	$C_{36}H_{51}O_{12}^+$	675.3375	675.3342	4.9
$C_{52}H_{71}O_{15}S^+$	967.4508	967.4441	6.9	$C_{36}H_{49}O_{11}^+$	657.3269	657.3299	-4.6
$C_{52}H_{79}O_{16}^+$	959.5363	959.5332	3.2	$C_{29}H_{43}O_{14}S^+$	647.2368	647.2335	5.1
$C_{52}H_{77}O_{15}^+$	941.5257	941.5212	4.8	$C_{36}H_{47}O_{10}^+$	639.3164	639.3132	5.0
$C_{52}H_{75}O_{14}^+$	923.5151	923.5134	1.8	$C_{29}H_{43}O_{11}^+$	567.2800	567.2772	4.9
$C_{52}H_{73}O_{13}^+$	905.5046	905.5016	3.3	$C_{29}H_{41}O_{10}^+$	549.2694	549.2696	-0.4
$C_{43}H_{65}O_{18}S^+$	901.3886	901.3838	5.3	$C_{23}H_{34}O_{3}^+$	357.2424	357.2404	5.6
$C_{52}H_{71}O_{12}^+$	887.4940	887.4904	4.1	$C_{23}H_{32}O_{2}^+$	339.2319	339.2286	9.7
$C_{43}H_{63}O_{17}S^+$	883.3786	881.3775	1.2	$C_{19}H_{27}O_{3}^+$	303.1960	303.1963	-1.0
$C_{52}H_{69}O_{11}^+$	869.4834	869.4818	1.8	$C_{16}H_{23}O_{2}^+$	247.1693	247.1689	1.6
$C_{52}H_{67}O_{10}^+$	851.4729	851.4692	4.3	$C_{15}H_{21}O_{2}^+$	233.1536	233.1523	5.6
$C_{39}H_{59}O_{17}S^+$	831.3467	831.3447	2.4	$C_{15}H_{19}O^+$	215.1430	215.1429	0.5
$C_{43}H_{65}O_{15}^+$	821.4318	821.4259	7.2	$C_{12}H_{17}O_{2}^+$	193.1223	193.1218	2.6
$C_{43}H_{63}O_{14}^+$	803.4212	803.4232	-2.5	$C_{12}H_{15}O^+$	175.1117	175.1107	5.7
$C_{43}H_{61}O_{13}^+$	785.4107	785.4105	0.3	$C_{8}H_{11}O^+$	123.0804	123.0800	3.2
$C_{43}H_{59}O_{12}^+$	767.4001	767.3981	2.6	$C_{7}H_{9}O^+$	109.0648	109.0640	7.3
$C_{39}H_{59}O_{14}^+$	751.3899	751.3905	-0.8	$C_{7}H_{11}^+$	95.0855	95.0848	7.4

Table S3. The proposed attributions of ion formulas along with mass differences (Δ ppm) of compound **4**

Elemental formula Ion	Theoretical *m*/*z*	Experimental *m*/*z*	Δ ppm	Elemental formula Ion	Theoretical *m*/*z*	Experimental *m*/*z*	Δ ppm
$C_{52}H_{81}O_{19}S^+$	1041.5087	1041.5110	-2.2	$C_{39}H_{61}O_{14}^+$	753.4056	753.4023	4.4
$C_{52}H_{79}O_{18}S^+$	1023.4982	1023.4875	0.7	$C_{39}H_{59}O_{13}^+$	735.3950	735.3922	3.8
$C_{52}H_{77}O_{17}S^+$	1005.4876	1005.4823	5.3	$C_{39}H_{57}O_{12}^+$	717.3845	717.3878	-4.6
$C_{52}H_{75}O_{16}S^+$	987.4770	987.4707	6.4	$C_{39}H_{53}O_{10}^+$	677.3532	677.3491	6.0
$C_{52}H_{73}O_{15}S^+$	969.4665	969.4724	-6.1	$C_{39}H_{51}O_{9}^+$	659.3426	659.3449	-3.5
$C_{52}H_{81}O_{16}^+$	961.5519	961.5490	3.0	$C_{29}H_{45}O_{14}S^+$	649.2525	649.2552	-4.2
$C_{52}H_{79}O_{15}^+$	943.5413	943.5385	3.0	$C_{29}H_{45}O_{11}^+$	569.2956	569.2946	1.8
$C_{52}H_{77}O_{14}^+$	925.5308	925.5295	1.4	$C_{29}H_{43}O_{10}^+$	551.2851	551.2836	2.7
$C_{52}H_{75}O_{13}^+$	907.5202	907.5202	0	$C_{23}H_{33}O_{3}^+$	357.2424	357.2414	2.8
$C_{43}H_{67}O_{18}S^+$	903.4043	903.4002	4.5	$C_{19}H_{27}O_{3}^+$	303.1955	303.1960	-1.6
$C_{52}H_{73}O_{12}^+$	889.5097	889.5150	-6.0	$C_{15}H_{21}O_{2}^+$	233.1536	233.1539	-1.3
$C_{43}H_{65}O_{17}S^+$	885.3937	885.3883	6.1	$C_{15}H_{19}O^+$	215.1430	215.1429	0.5
$C_{52}H_{71}O_{11}^+$	871.4991	871.4918	8.4	$C_{12}H_{17}O_{2}^+$	193.1223	193.1232	-4.7
$C_{52}H_{69}O_{10}^+$	853.4885	853.4848	4.3	$C_{12}H_{15}O^+$	175.1117	175.1113	2.3
$C_{39}H_{61}O_{17}S^+$	833.3624	833.3630	-0.7	$C_{8}H_{11}O^+$	123.0804	123.0805	-0.8
$C_{43}H_{67}O_{15}^+$	823.4474	823.4414	7.3	$C_{7}H_{9}O^+$	109.0648	109.0655	-6.4
$C_{43}H_{65}O_{14}^+$	805.4369	805.4351	2.2	$C_{7}H_{11}^+$	95.0855	95.0851	4.2
$C_{43}H_{63}O_{13}^+$	787.4263	787.4219	5.6				

Table S4. The proposed attributions of ion formulas along with mass differences (Δ ppm) of compound **3**

Elemental formula Ion	Theoretical *m/z*	Experimental *m/z*	Δ ppm	Elemental formula Ion	Theoretical *m/z*	Experimental *m/z*	Δ ppm
$C_{52}H_{79}O_{18}S^+$	1023.4982	1023.5005	-2.2	$C_{36}H_{55}O_{13}^+$	695.3637	695.3593	6.3
$C_{52}H_{77}O_{17}S^+$	1005.4876	1005.4837	3.9	$C_{36}H_{53}O_{12}^+$	677.3532	677.3514	2.7
$C_{52}H_{75}O_{16}S^+$	987.4770	987.4820	-5.1	$C_{39}H_{51}O_9^+$	659.3426	659.3394	4.8
$C_{52}H_{79}O_{15}^+$	943.5413	943.5415	-0.2	$C_{29}H_{45}O_{11}^+$	569.2956	569.2905	9.0
$C_{52}H_{77}O_{14}^+$	925.5308	925.5317	-1.0	$C_{23}H_{33}O_3^+$	357.2424	357.2407	4.8
$C_{52}H_{75}O_{13}^+$	907.5202	907.5225	-2.5	$C_{19}H_{27}O_3^+$	303.1955	303.1961	-2.0
$C_{52}H_{73}O_{12}^+$	889.5097	889.5058	4.4	$C_{18}H_{25}O^+$	257.1900	257.1884	6.2
$C_{47}H_{65}O_{14}S^+$	885.3937	885.3916	2.4	$C_{15}H_{21}O_2^+$	233.1536	233.1529	3.0
$C_{52}H_{71}O_{11}^+$	871.4991	871.4957	3.9	$C_{15}H_{19}O^+$	215.1430	215.1421	4.2
$C_{52}H_{69}O_{10}^+$	853.4885	853.4925	-4.7	$C_{12}H_{17}O_2^+$	193.1223	193.1224	-0.5
$C_{39}H_{59}O_{16}S^+$	815.3518	815.3545	-3.3	$C_{13}H_{17}O^+$	189.1274	189.1269	2.6
$C_{43}H_{65}O_{14}^+$	805.4369	805.4335	4.2	$C_{12}H_{15}O^+$	175.1117	175.1116	0.6
$C_{43}H_{63}O_{13}^+$	787.4263	787.4207	7.1	$C_{12}H_{13}^+$	157.1012	157.1022	-6.4
$C_{43}H_{61}O_{12}^+$	769.4158	769.4211	-6.9	$C_8H_{11}O^+$	123.0804	123.0804	0
$C_{39}H_{59}O_{13}^+$	735.3950	735.3932	2.4	$C_7H_9O^+$	109.0648	109.0641	6.4
$C_{39}H_{57}O_{12}^+$	717.3845	717.3821	3.3	$C_7H_{11}^+$	95.0855	95.0858	-3.2

Table S5. The proposed attributions of ion formulas along with mass differences (Δ ppm) of compound **6**

Elemental formula Ion	Theoretical *m*/*z*	Experimental *m*/*z*	Δ ppm	Elemental formula Ion	Theoretical *m*/*z*	Experimental *m*/*z*	Δ ppm
$C_{52}H_{77}O_{18}S^+$	1021.4825	1021.4759	6.5	$C_{36}H_{47}O_{10}^+$	639.3158	639.3149	1.4
$C_{52}H_{75}O_{17}S^+$	1003.4719	1003.4662	5.7	$C_{23}H_{33}O_3^+$	357.2424	357.2404	5.6
$C_{52}H_{73}O_{16}S^+$	985.4614	985.4577	3.8	$C_{19}H_{25}O_2^+$	285.1849	285.1834	5.3
$C_{52}H_{77}O_{15}^+$	941.5257	941.5198	6.3	$C_{18}H_{25}O^+$	257.1900	257.1895	1.9
$C_{52}H_{75}O_{14}^+$	923.5151	923.5115	3.9	$C_{16}H_{23}O_2^+$	247.1693	247.1693	0
$C_{52}H_{73}O_{13}^+$	905.5046	905.5006	4.4	$C_{15}H_{21}O_2^+$	233.1536	233.1518	7.7
$C_{52}H_{71}O_{12}^+$	887.4940	887.4880	6.8	$C_{15}H_{19}O^+$	215.1430	215.1436	-2.8
$C_{43}H_{63}O_{17}S^+$	883.3780	883.3728	5.9	$C_{13}H_{19}O^+$	191.1430	191.1421	4.7
$C_{52}H_{69}O_{11}^+$	869.4834	869.4807	3.1	$C_{11}H_{13}O^+$	161.0961	161.0966	-3.1
$C_{52}H_{67}O_{10}^+$	851.4729	851.4651	9.2	$C_{10}H_{13}O^+$	149.0961	149.0947	9.4
$C_{52}H_{65}O_9^+$	833.4623	833.4628	-0.6	$C_8H_{11}O^+$	123.0804	123.0816	-9.7
$C_{39}H_{57}O_{16}S^+$	813.3362	813.3280	10.1	$C_7H_9O^+$	109.0648	109.0645	2.8
$C_{43}H_{63}O_{14}^+$	803.4212	803.4153	7.3	$C_6H_9^+$	81.0699	81.0692	8.6
$C_{36}H_{53}O_{13}^+$	693.3481	693.3437	6.3				

Table S6. The proposed attributions of ion formulas along with mass differences (Δ ppm) of compound **2**

Elemental formula Ion	Theoretical *m/z*	Experimental *m/z*	Δ ppm	Elemental formula Ion	Theoretical *m/z*	Experimental *m/z*	Δ ppm
$C_{51}H_{82}NO_{17}^{+}$	980.5583	980.5568	1.5	$C_{43}H_{65}O_{15}^{+}$	821.4318	821.4287	3.8
$C_{51}H_{79}O_{17}^{+}$	963.5312	963.5321	-0.9	$C_{44}H_{67}O_{14}^{+}$	819.4525	819.4484	5.0
$C_{51}H_{77}O_{16}^{+}$	945.5206	945.5161	4.8	$C_{46}H_{67}O_{12}^{+}$	811.4627	811.4594	4.1
$C_{51}H_{75}O_{15}^{+}$	927.5100	927.5068	3.5	$C_{44}H_{63}O_{10}^{+}$	751.4416	751.4348	9.0
$C_{51}H_{73}O_{14}^{+}$	909.4995	909.4960	3.8	$C_{44}H_{61}O_{9}^{+}$	733.4310	733.4279	4.2
$C_{51}H_{71}O_{13}^{+}$	891.4889	891.4876	1.5	$C_{18}H_{25}O_{3}^{+}$	289.1798	289.1776	7.6
$C_{51}H_{69}O_{12}^{+}$	873.4784	873.4753	3.5	$C_{14}H_{19}O_{2}^{+}$	219.1380	219.1371	4.1
$C_{51}H_{67}O_{11}^{+}$	855.4678	855.4679	-0.1	$C_{11}H1_{3}O^{+}$	161.0961	161.0954	4.3
$C_{51}H_{65}O_{10}^{+}$	837.4572	837.4513	7.0	$C_{7}H_{9}O^{+}$	109.0648	109.0643	4.6
$C_{46}H_{69}O_{13}^{+}$	829.4733	829.4663	8.4	$C_{6}H_{9}^{+}$	81.0699	81.0691	9.9

Table S7. Mass spectrometer conditions for analysis of gambierone and 44-methylgambierone

compound	Parent ion (*m/z*)	Product ion (*m/z*)	DP (V)	EP (V)	CE (V)	CXP (V)
gambierone	1023.407	96.700	-5	-10	-130	-13
44-methylgambierone	1037.507	96.700	-5	-10	-106	-31

Disclaimer/Publisher's Note: The statements, opinions and data contained in all publications are solely those of the individual author(s) and contributor(s) and not of MDPI and/or the editor(s). MDPI and/or the editor(s) disclaim responsibility for any injury to people or property resulting from any ideas, methods, instructions or products referred to in the content.
